# Emergence of dominant toxigenic M1T1 *Streptococcus pyogenes* clone during increased scarlet fever activity in England: a population-based molecular epidemiological study

**DOI:** 10.1016/S1473-3099(19)30446-3

**Published:** 2019-11

**Authors:** Nicola N Lynskey, Elita Jauneikaite, Ho Kwong Li, Xiangyun Zhi, Claire E Turner, Mia Mosavie, Max Pearson, Masanori Asai, Ludmila Lobkowicz, J Yimmy Chow, Julian Parkhill, Theresa Lamagni, Victoria J Chalker, Shiranee Sriskandan

**Affiliations:** a Department of Infectious Diseases and Medical Research Council Centre for Molecular Bacteriology & Infection, Imperial College London, London, UK; b Department of Infectious Disease Epidemiology, School of Public Health, Imperial College London, London, UK; c Health Protection Research Unit in Healthcare Associated Infections and Antimicrobial Resistance, National Institute for Health Research, Imperial College London, London, UK; d Molecular Biology & Biotechnology, University of Sheffield, Sheffield, UK; e North-West London Health Protection Team, London Public Health England Centre, Public Health England, London, UK; f National Infection Service, Public Health England, London, UK; g Wellcome Sanger Institute, Cambridge, UK

## Abstract

**Background:**

Since 2014, England has seen increased scarlet fever activity unprecedented in modern times. In 2016, England's scarlet fever seasonal rise coincided with an unexpected elevation in invasive *Streptococcus pyogenes* infections. We describe the molecular epidemiological investigation of these events.

**Methods:**

We analysed changes in *S pyogenes emm* genotypes, and notifications of scarlet fever and invasive disease in 2014–16 using regional (northwest London) and national (England and Wales) data. Genomes of 135 non-invasive and 552 invasive *emm*1 isolates from 2009–16 were analysed and compared with 2800 global *emm*1 sequences. Transcript and protein expression of streptococcal pyrogenic exotoxin A (SpeA; also known as scarlet fever or erythrogenic toxin A) in sequenced, non-invasive *emm*1 isolates was quantified by real-time PCR and western blot analyses.

**Findings:**

Coincident with national increases in scarlet fever and invasive disease notifications, *emm*1 *S pyogenes* upper respiratory tract isolates increased significantly in northwest London in the March to May period, from five (5%) of 96 isolates in 2014, to 28 (19%) of 147 isolates in 2015 (p=0·0021 *vs* 2014 values), to 47 (33%) of 144 in 2016 (p=0·0080 *vs* 2015 values). Similarly, invasive *emm*1 isolates collected nationally in the same period increased from 183 (31%) of 587 in 2015 to 267 (42%) of 637 in 2016 (p<0·0001). Sequences of *emm*1 isolates from 2009–16 showed emergence of a new *emm*1 lineage (designated M1_UK_)—with overlap of pharyngitis, scarlet fever, and invasive M1_UK_ strains—which could be genotypically distinguished from pandemic *emm*1 isolates (M1_global_) by 27 single-nucleotide polymorphisms. Median SpeA protein concentration in supernatant was nine-times higher among M1_UK_ isolates (190·2 ng/mL [IQR 168·9–200·4]; n=10) than M1_global_ isolates (20·9 ng/mL [0·0–27·3]; n=10; p<0·0001). M1_UK_ expanded nationally to represent 252 (84%) of all 299 *emm*1 genomes in 2016. Phylogenetic analysis of published datasets identified single M1_UK_ isolates in Denmark and the USA.

**Interpretation:**

A dominant new *emm*1 *S pyogenes* lineage characterised by increased SpeA production has emerged during increased *S pyogenes* activity in England. The expanded reservoir of M1_UK_ and recognised invasive potential of *emm*1 *S pyogenes* provide plausible explanation for the increased incidence of invasive disease, and rationale for global surveillance.

**Funding:**

UK Medical Research Council, UK National Institute for Health Research, Wellcome Trust, Rosetrees Trust, Stoneygate Trust.

## Introduction

Scarlet fever is a classic exanthem of childhood caused by the bacterium *Streptococcus pyogenes* (group A streptococcus) that, until the beginning of the 20th century, was associated with frequent loss of life among children.^1^ By the start of the 20th century, long before widespread use of antibiotics, the incidence and severity of scarlet fever had begun to fall, a phenomenon that remains largely unexplained.^2^ One potential (untestable) hypothesis is that the streptococcal bacteria causing the disease might have undergone a pathogenetic change that led to a reduction in the invasive and septic sequelae of scarlet fever.

Since the 1940s, scarlet fever has followed a seasonal springtime pattern—peaking between March and May while remaining less frequent throughout the rest of the year—without the major cyclical epidemics observed in the early 20th century.^3^ Surges in invasive infections can periodically follow a similar seasonal pattern for reasons that are incompletely understood. In 2014, England had an unexpected surge in scarlet fever infections, with over 15 000 disease notifications—a marked increase in incidence compared with previous decades.^3^, ^4^ Despite having a major impact on public health resources,^3^ the increase in infections was not associated with any rise in the incidence of invasive disease. Even greater seasonal upsurges of scarlet fever were observed in 2015, when there were over 17 000 notifications, and in 2016, when there were over 19 000 notifications.^3^ In the spring of 2016, there was a 1·5-times increase in the number of laboratory-confirmed invasive *S pyogenes* infections compared with that in the previous 5 years, coinciding with the peak in scarlet fever notifications.^3^, ^5^ The absence of any association between scarlet fever notifications and increased invasive infection notifications in 2014^3^ led us to speculate that the association of scarlet fever with invasive disease in 2016 might be strain dependent.

**Research in context**

**Evidence before this study**

In March to May of 2016, an unexpected elevation in notifications of invasive *Streptococcus pyogenes* infections in England was seen, coinciding with a national increase in notifications of seasonal scarlet fever (a paediatric exanthem also caused by *S pyogenes*). Since 2014, scarlet fever notifications in England have reached unexpectedly high levels, peaking between March and May each year, although notifications of invasive *S pyogenes* infections in 2014 were within expected limits, in contrast to 2016. We aimed to test the hypothesis that the link between scarlet fever and invasive infection patterns might be strain-related and, in the process, identified the emergence of a new M1T1 lineage. We searched PubMed for clinical and laboratory studies published before March 1, 2019, using the search terms “scarlet fever” and “upsurge” or “mortality”, as well as “*emm*1” or “M1T1” and “streptococcus”. We also searched using the terms “SpeA” or “scarlet fever toxin” or “erythrogenic toxin” and “streptococcus” and “regulation”. We identified studies describing recent and historical trends in scarlet fever incidence, studies that described trends in strain types causing invasive infections, and studies that linked SpeA to dominant strains. We also found studies of toxin expression that reported links with phage induction, growth phase regulation, transcriptional regulators, proteolysis, and host proteins as potential regulators.

**Added value of this study**

Our study provides a molecular explanation for the association between increased incidence of scarlet fever and increased incidence of invasive *S pyogenes* infections, by identifying an emergent lineage of M1T1 *S pyogenes* (M1_UK_) that expanded rapidly to become the largest single contributor to both non-invasive and invasive infections in 2016. The findings raise the possibility that historical associations between epidemic waves of scarlet fever and invasive infections might also have been linked to strain pathogenicity, in addition to general population susceptibility. Genomic analysis confirmed that the strains that cause scarlet fever are no different to those that cause streptococcal pharyngitis and rarer invasive infections. Increases in one disease could lead to increases in all, particularly if the lineage involved is highly pathogenic. The emergent lineage was characterised by a number of genetic changes that were predictive of increased production of SpeA, and this increased production was confirmed by laboratory testing. Although this might be just one of many changes in the new lineage, increased production of SpeA is predicted to enhance bacterial fitness, as suggested by the increasing dominance of the new lineage in comparison to older M1T1 strains in England. The work highlights that group A streptococcal lineages can differ in pathogenicity.

**Implications of all the available evidence**

Scarlet fever notifications in England in the period 2014–18 are the highest seen since 1960, and incidence in young children exceeds that reported in other countries. It is uncertain whether the increase in scarlet fever is a result of practice change or other population or environmental factors; the new lineage described is not implicated in the initial upsurge. However, there is a need to curtail this increasing trend. Interventions targeted at the population at risk have the potential to reduce the reservoir of *S pyogenes* that can seed more harmful invasive infections. Research to identify the most appropriate intervention is underway and practice guidelines for streptococcal pharyngitis might need to take strain variation and wider population effects into account. Increased *S pyogenes* disease activity could provide a platform for strain evolution and expansion, highlighting an unforeseen consequence of modern epidemics. The genetic changes in the emergent M1T1 clone require detailed laboratory investigation to understand the wider phenotypic changes that have occurred and the molecular basis for these, including transmissibility and response to treatment. It is not known whether the new lineage will recede in due course, as other lineages have done, or if it will remain dominant in the population, and surveillance is needed. Detection of new lineage representatives outside the UK underlines a need for global surveillance and increased vigilance if strains with increased fitness and altered phenotype are detected.

Microbiological surveillance of *S pyogenes* upper respiratory tract infections in England is constrained, as most doctors do not routinely take samples for bacteriological diagnosis of sore throat. However, all *S pyogenes* isolates cultured from samples submitted from the population of northwest London are collected and archived by the Imperial College Infection Biobank, allowing longitudinal study of strains causing both non-invasive and invasive infections. Alongside the national reference laboratory, which systematically archives invasive *S pyogenes* isolates from across the country, the collection provides unique insight into the relationship between upper respiratory tract isolates and isolates from rare, but more severe and invasive, infections.

*S pyogenes* can be serotyped or genotyped on the basis of the M antigen, which is encoded by the *emm* gene. Changes in disease incidence are sometimes characterised by expansion of specific *emm* genotypes.^4^ Using a combination of epidemiological and bacteriological approaches, we set out to identify the *emm* genotypes responsible for *S pyogenes* infections during the 2014–16 scarlet fever seasons regionally, then extended the study nationally, to identify bacterial determinants that might explain the observed increase in invasive disease due to *S pyogenes* in 2016.

## Methods

### *S pyogenes* notifications

Cases of suspected scarlet fever are notified by clinicians to Public Health England on the basis of symptoms and signs consistent with scarlet fever, with or without laboratory confirmation of *S pyogenes* infection. Scarlet fever has been notifiable since 1899 in England. Since 2010, cases of invasive *S pyogenes* infections have also been notifiable to Public Health England, in accordance with statutory regulations.

### *S pyogenes* isolates and *emm* genotyping

All non-invasive *S pyogenes* isolates submitted to the Diagnostic Laboratory at Imperial College Healthcare National Health Service Trust (London, UK) between Jan 1, 2009 and Dec 31, 2013, and between March 1 and May 31 each year during 2014–16 were cultured and stored frozen in glycerol (sampling rationale is detailed in the appendix p 29). This laboratory serves northwest London, a population of roughly 2 million people, representing approximately 3% of the population of England. Clinical data were linked to isolates and anonymised in accordance with research ethics approval (number 06/Q0406/20). Isolates were *emm* genotyped (appendix p 29). Laboratories from England and Wales are requested to submit sterile site and invasive *S pyogenes* isolates to the national reference laboratory, where *emm* genotyping is done on all submitted isolates. All isolates were cultured on Columbia blood agar (Oxoid, Basingstoke, UK) or in Todd-Hewitt broth (Oxoid) at 37°C with 5% CO_2_.

### Genome sequencing

All non-invasive *emm*1 *S pyogenes* isolates collected from northwest London from 2009 to 2016 were subject to genome sequencing (appendix p 29), as were invasive *emm*1 isolates collected from England and Wales from March to May of 2013 and 2016. Comparative genomic analysis of invasive isolates was done with new (2013 and 2016) and existing (2014–15)^6^ genome sequence data (appendix p 12–27). DNA from *S pyogenes* isolates was prepared, sequenced, analysed, and compared with published data from the UK (2007–12), North America, Nordic regions, and southeast Asia^6^, ^7^, ^8^, ^9^, ^10^, ^11^ (appendix pp 28–29). All new genome sequence data generated in this study have been submitted to the European Nucleotide Archive) under the accession numbers listed in the appendix (pp 8, 12).

### Quantification of *speA* production

Transcript expression of *speA,* encoding streptococcal pyrogenic exotoxin A (SpeA; also known as scarlet fever or erythrogenic toxin A), was quantified by real-time PCR using a standard curve method. SpeA protein expression in 5 × concentrated overnight supernatants was assessed with western blotting, and compared with a recombinant SpeA standard (appendix pp 29, 30). For SpeA protein testing, one or two isolates per year (2009–15) per lineage (M1_global_ *vs* M1_UK_) were randomly selected by lot, after excluding any strains with mutations in the two-component regulator *covRS* (also known as *csrRS*), which is known to repress a number of virulence factors, including SpeA.^12^

### Statistical analysis

Datasets were compared with a two-tailed Mann Whitney *U* test for continuous data or a χ^2^ test for categorical data, using GraphPad Prism 5.0 software. p values less than 0·05 were considered to indicate statistical significance. To assess trends in invasive disease incidence, Poisson regression in Stata (version 15) was done using mid-year population denominators for England from the Office for National Statistics.

### Role of the funding source

The funders of the study had no role in study design, data collection, data analysis, data interpretation, or writing of the report. The corresponding author had full access to all the data in the study and had final responsibility for the decision to submit for publication.

## Results

**Figure 1:**
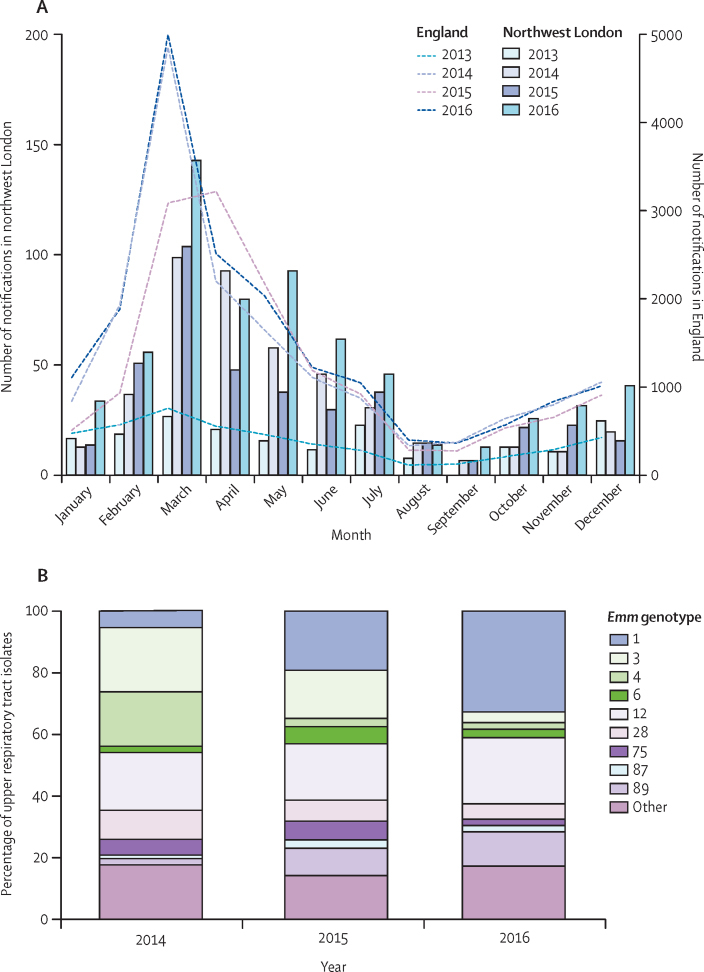
2016 surge in scarlet fever associated with expansion of *emm*1 upper respiratory tract isolates of *Streptococcus pyogenes* (A) Monthly notifications of scarlet fever in northwest London (bars) in 2013–16 showing the surge in notifications between March and May, peaking in 2016. National scarlet fever notifications (dashed lines) are shown for comparison. (B) *emm* genotyping of all upper respiratory tract isolates of *S pyogenes* from northwest London between March and May each year during 2014–16. *emm*1 strains emerged as the dominant upper respiratory tract genotype by 2016.

The northwest London population had a year-on-year rise in scarlet fever notifications between 2014 and 2016 that was representative of the country as a whole when compared with national notification data (figure 1A). We also compared notifications of invasive *S pyogenes* infections in northwest London with national data (appendix p 2), which showed a marked increase in *S pyogenes* invasive disease in 2016 in northwest London during the scarlet fever season that also mirrored the national pattern of increased notifications in 2016.

*emm* genotypes of all *S pyogenes* upper respiratory tract isolates obtained in northwest London during the scarlet fever seasons spanning 2015 and 2016 were ascertained and compared with existing data from 2014.^4^ *emm*1 upper respiratory tract strains increased in frequency year-on-year in the scarlet fever seasons, from five (5%) of 96 isolates in 2014, to 28 (19%) of 147 isolates in 2015 (p=0·0021 *vs* 2014 value), and 47 (33%) of 144 in 2016 (p=0·0080 *vs* 2015 value); in 2016, *emm*1 became the single most frequent *S pyogenes* genotype causing upper respiratory tract infections (figure 1B). The increase in *emm*1 strains in 2016 contrasted with the previously reported associations of *emm*3 and *emm*4 strains with the initial upsurge in scarlet fever in 2014.^4^ Brief clinical details were supplied for 243 of 387 upper respiratory tract isolates overall, among which 53 (22%) mentioned scarlet fever. Of these scarlet fever-associated isolates, those genotyped as *emm*1 increased from zero (0%) of 17 in 2014, to four (24%) of 17 in 2015, and seven (37%) of 19 in 2016 (p=0·0053 *vs* 2014 value).

**Figure 2:**
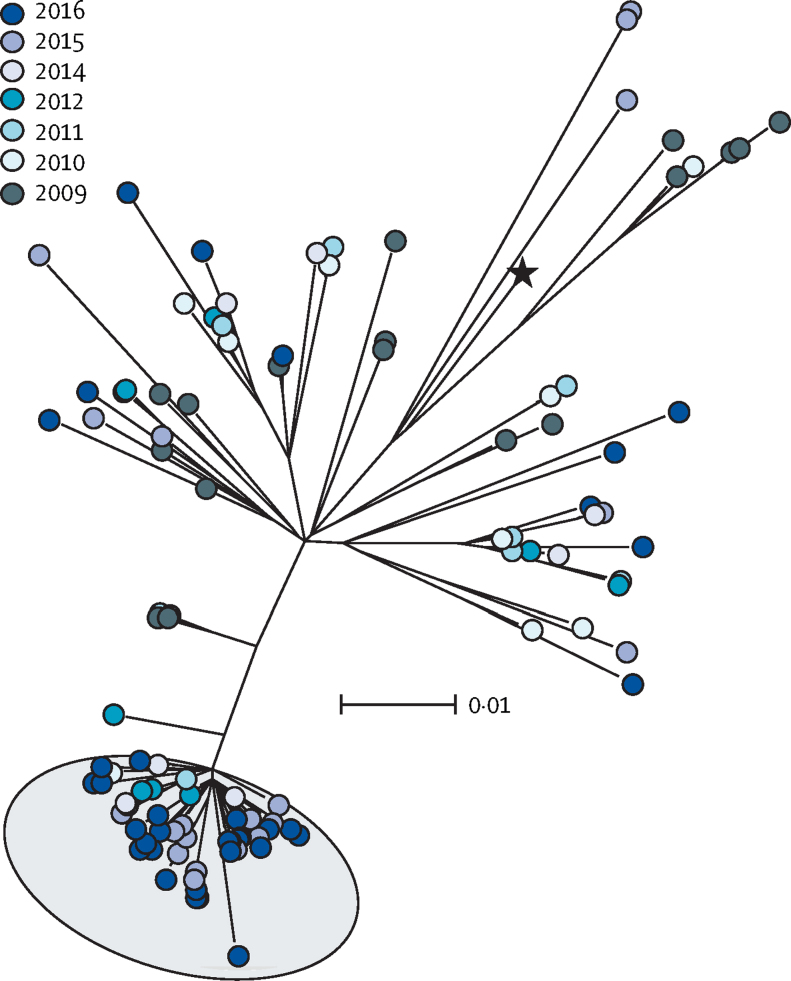
Emergence of new *emm*1 lineage among non-invasive *Streptococcus pyogenes* isolates Maximum likelihood phylogenetic tree constructed from core single-nucleotide polymorphisms (excluding prophage regions) of *emm*1 non-invasive isolates collected in northwest London in 2009–16 (n=135). Background shading in grey indicates the emergent lineage M1_UK_. 52 (85%) of 61 strains within the emergent lineage were isolated in either 2015 or 2016. The scale bar indicates the nucleotide substitutions per site. The black star indicates the reference strain MGAS5005. See appendix (p 3) for rooted tree with available metadata.

To identify any genetic basis for the expansion of *emm*1 *S pyogenes* among upper respiratory tract isolates collected in London, the genomes of all non-invasive *emm*1 isolates available from northwest London from 2009 to 2016 were sequenced (n=135). Single-nucleotide polymorphism (SNP)-based analysis of *emm*1 strains pointed to the emergence of a new *emm*1 lineage (designated M1_UK_), which could be differentiated from the contemporary *emm*1 population (M1_global_) by the presence of 27 core SNPs in regulatory and metabolic genes (figure 2, appendix p 7). The earliest member of the M1_UK_ lineage was identified in 2010, and five intermediate isolates (with 13 or 23 of the unique SNPs) were detected between 2009 and 2012 (appendix p 3). Similarly to M1_global_, M1_UK_ isolates were identified among all age groups, but included more cases of scarlet fever and evidence of recent transmissions than M1_global_ (appendix p 3). From 2015 onwards, around two-thirds of non-invasive *emm*1 isolates from northwest London were within the new M1_UK_ lineage (22 [71%] of 31 isolates in 2015, and 30 [65%] of 46 in 2016). Recombination and pan-genome analyses provided no evidence for gain or loss of transferable elements when comparing M1_global_ and M1_UK_ strains. Lineage-specific acquisition of antimicrobial-resistance genes was not detected; evidence of the *mefA* and *msrD* macrolide-resistance locus was found in just one M1_UK_ isolate, while eight M1_global_ isolates possessed antimicrobial-resistance genes (one isolate with the *mefA* and *msrD* locus, one with the tetracycline-resistance gene *tetM*, and six with the aminoglycoside-resistance gene *aph3*).

**Figure 3:**
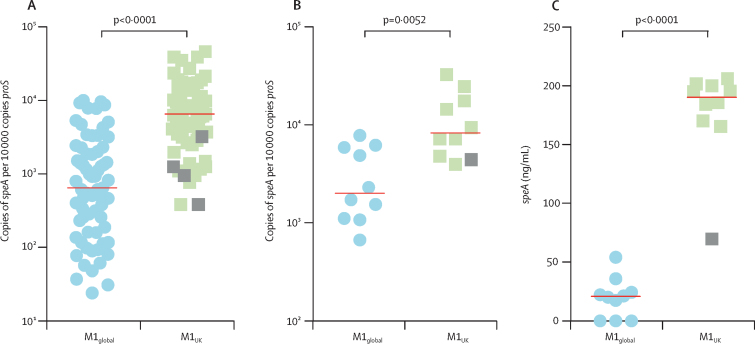
Expression of scarlet fever toxin SpeA in emergent M1_UK_ isolates and other *emm*1 isolates Phenotypic comparison of emergent M1_UK_ isolates with M1_global_ strains. (A) Quantification of absolute copy number of *speA* transcripts relative to house-keeping gene *proS* in all genome-sequenced non-invasive isolates from northwest London (n=135). Isolates were assigned to each lineage on the basis of SNPs (appendix pp 7–11). Quantification of *speA* transcript expression (B) and SpeA protein concentration in supernatant (C) from randomly selected M1_UK_ and M1_global_ strains lacking any mutations in *covRS* (n=10 per group). Median values are shown by horizontal lines in each graph. p values are from Mann-Whitney *U* tests. Grey squares denote strains that are intermediate members of the M1_UK_ lineage. SpeA=streptococcal pyrogenic exotoxin type A.

Scarlet fever is a toxin-mediated syndrome, historically associated with the expression of the phage-encoded erythrogenic toxin SpeA,^13^ the gene for which is possessed by all *emm*1 isolates studied in this investigation. Among the 27 M1_UK_ lineage-defining SNPs, three non-synonymous mutations were identified in the gene *rofA* encoding the standalone transcriptional regulator RofA,^14^ which, alongside a homologue, *nra*, has been implicated as a repressor of SpeA production in some, but not all, streptococcal genotypes.^15^, ^16^ Real-time PCR analysis of all sequenced non-invasive *emm*1 isolates from northwest London (n=135) indicated that the emergent lineage (M1_UK_) was associated with significantly increased transcription of *speA* compared with other contemporary *emm*1 isolates (M1_global_), suggesting that differential SpeA production was a feature of the M1_UK_ lineage (figure 3A).

To ascertain whether this difference in transcription translated into a difference in SpeA protein expression, we compared ten M1_UK_ (including one intermediate strain harbouring only 13 of the 27 lineage-defining SNPs) and ten M1_global_ isolates and corroborated our finding that M1_UK_ isolates had enhanced *speA* transcription (figure 3B). Quantitative western blot analysis of SpeA protein in bacterial overnight culture supernatant showed nine-times greater median SpeA production in the emergent M1_UK_ lineage (190·2 ng/mL [IQR 168·9–200·4]) than in the M1_global_ strains (20·9 ng/mL [0·0–27·3]; p<0·0001; figure 3C). The M1_UK_ strain representing an intermediate genotype (13 of 27 SNPs, including the *rofA* SNPs) did not show enhanced SpeA protein secretion (figure 3C).

The sequence for *speA* in non-invasive M1_UK_ and M1_global_ isolates was identical to that of the reference strain MGAS5005. Integration sites for the phage carrying *speA* in sequenced non-invasive M1_UK_ and M1_global_ isolates were consistent with those in the reference strain, and mapped sequences from M1_UK_ and M1_global_ isolates were very similar (if not identical) to the reference strain phage 5005.1; no evidence of translocated superantigens or duplication of *speA* was found.

**Figure 4:**
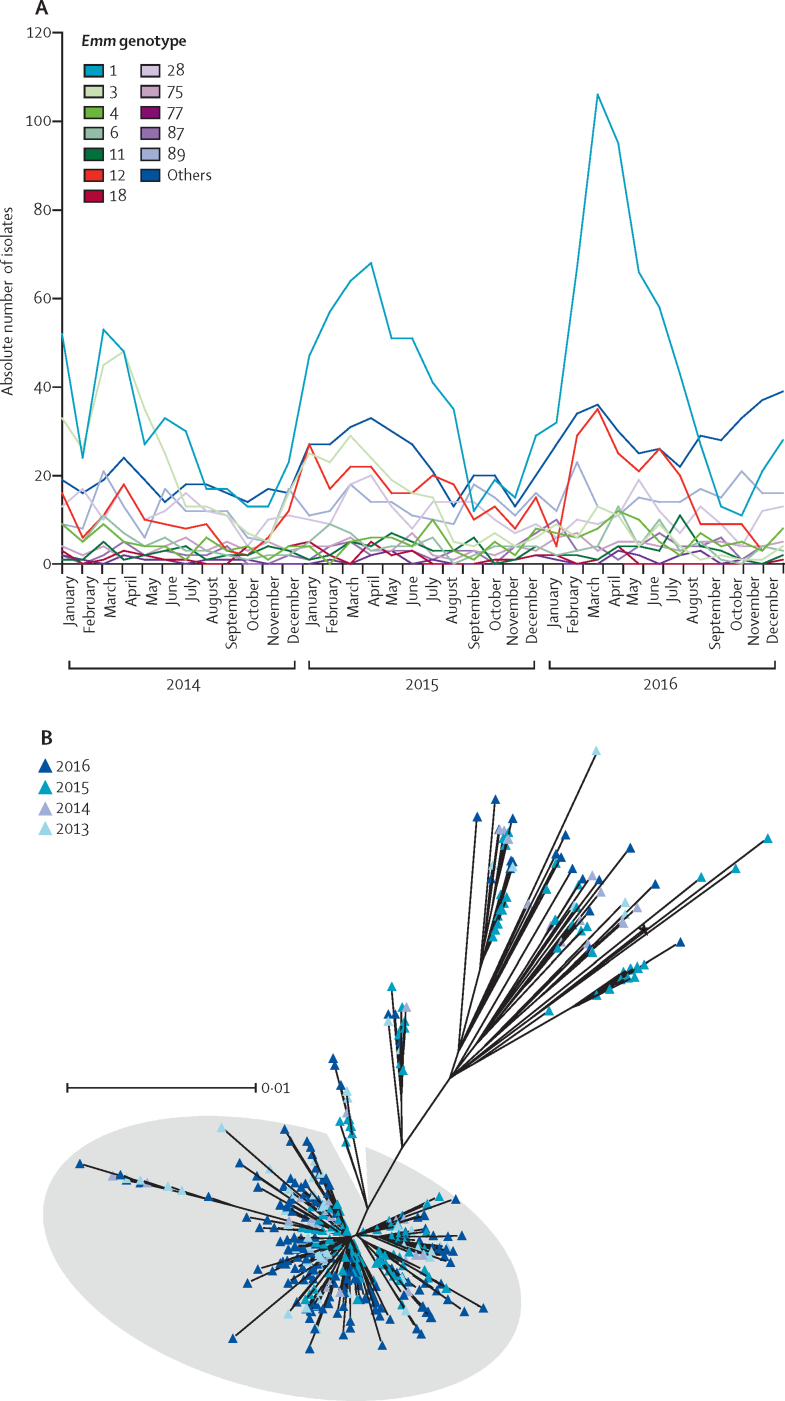
Prevalence of *emm*1 strains among invasive *Streptococcus pyogenes* infections nationally during scarlet fever seasons and emergence of M1_UK_ lineage over time (A) *emm* genotypes of invasive *S pyogenes* isolates referred to national reference laboratory per month during 2014–16. Increases in total invasive disease cases were observed locally and nationally during March to May 2016 (appendix p 2). (B) Maximum likelihood phylogenetic tree constructed from core single-nucleotide polymorphisms (excluding prophage regions) of genome-sequenced invasive *emm*1 *S pyogenes* isolates in England and Wales (n=552) between March and May each year during 2013–16. Shading in grey indicates the emergent lineage M1_UK_. Clustering was not observed based on geographical origin, indicating emergence of the lineage on a national level. The black star indicates the reference strain MGAS5005. The scale bar represents the number of nucleotide substitutions per site.

Although there was no rise in invasive disease notifications in 2014, the first year of elevated scarlet fever activity, a marked increase was seen nationally in the spring of 2016 compared with the same period (March to May) in the preceding 3 years (rate ratio 1·43 [95% CI 1·31–1·56], p<0·001; appendix p 2). Although invasive disease case numbers in northwest London were modest when considered on a monthly basis by comparison with national data (appendix p 2), the seasonal increase in notifications matched that observed nationally. *emm* genotyping of all invasive disease isolates referred to the national laboratory showed significant absolute and relative year-on-year increases in *emm*1, from 183 (31%) of 587 isolates between March and May 2015, to 267 (42%) of 637 in the same period in 2016 (p<0·0001 *vs* 2015 values), peaking March 2016 (figure 4A).

To ascertain whether invasive *S pyogenes* infections might be affected by the emergent M1_UK_ lineage, we compared the genome sequences of 552 invasive *emm*1 isolates (appendix p 12–27) in England and Wales from March to May each year during 2013–16. Focusing first on London, where sequence data from non-invasive isolates were available for comparison, SNP-based phylogenetic analysis showed intermingling of the 31 sterile-site invasive and 135 non-invasive isolates; 16 (84%) of 19 invasive strains obtained in 2015 and 2016 lay within the emergent M1_UK_ lineage, compared with just five (42%) of 12 obtained between 2013 and 2014 (appendix p 4). Four (13%) of 31 invasive isolates were identical to, or no more than two SNPs different from, non-invasive isolates in the community, consistent with recent transmission.

**Figure 5:**
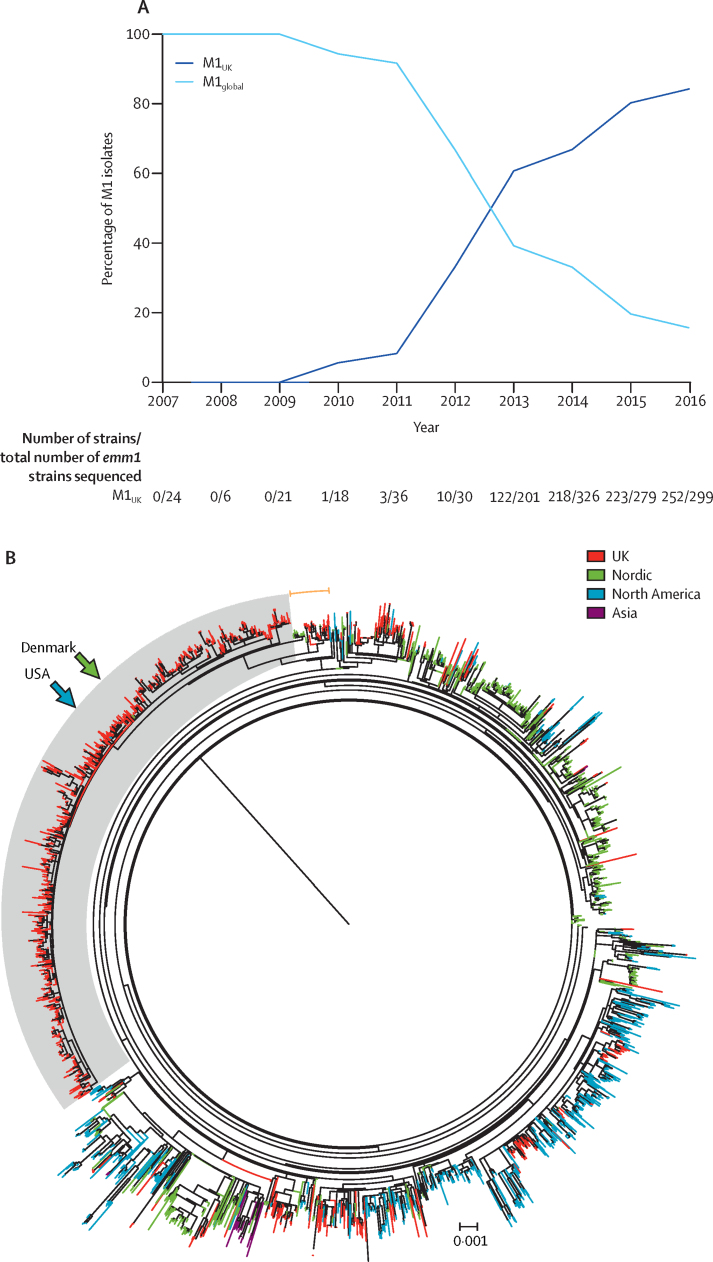
Longitudinal (A) and geographical (B) comparison of M1_UK_ lineage with pandemic *emm*1 strains of *Streptococcus pyogenes* (A) Proportions of M1_UK_ and M1_global_ isolates among total sequenced invasive and non-invasive *emm*1 *S pyogenes* isolates (n=1240) annually in the UK between 2007 and 2016. (B) M1_UK_ lineage in a global context. Maximum likelihood phylogenetic tree constructed from core SNPs (excluding prophage regions) comparing all sequenced UK *emm*1 isolates with the global *emm*1 populations from North America, Nordic countries, and Asia (n=2800 isolates). Shading in grey indicates the emergent lineage M1_UK_; orange arc indicates intermediate isolates that lie outside M1_UK_ but possess 13 or more of the 27 SNPs present in M1_UK_, including three SNPs in *rofA*. UK and international *emm*1 isolates arise throughout the tree, but isolates within the M1_UK_ lineage are exclusively from the UK, except two single isolates from Denmark and the USA (arrows). The scale bar indicates the number of nucleotide substitutions per site. See appendix (p 6) for the unrooted tree. SNPs=single-nucleotide polymorphisms.

We then analysed the genome sequences of all 552 *emm*1 sterile site isolates collected between March and May each year from 2013 to 2016, obtained nationally from across different geographical locations in England and Wales. 425 (77%) of 552 invasive *emm*1 strains were within the new lineage (figure 4B), which was present in the UK invasive isolate population as early as 2013. Like M1_global_ strains, M1_UK_ strains were phylogenetically distinct from the historical UK *emm*1 scarlet fever *speA*-positive isolate NCTC8198 and *emm*1 *speC*-positive SF370 (appendix p 5). Longitudinal analysis of all available 1240 UK *emm*1 sequences (appendix p 28)^6^, ^7^, ^8^ obtained from invasive and non-invasive disease cases showed a yearly increase in M1_UK_ such that, by 2016, M1_UK_ strains represented more than 80% of all available *emm*1 isolates in the UK, outnumbering M1_global_ strains (figure 5A).

Phylogenetic comparison of UK *emm*1 sequences with available international sequences from North America, Nordic regions, UK, and southeast Asia (appendix p 28)^9^, ^10^, ^11^ confirmed that M1_UK_ strains were distinct from the globally disseminated pandemic *emm*1 strains (figure 5B). A small number of intermediate isolates (possessing at least 13 of the 27 lineage-defining SNPs, including three SNPs in *rofA*) were identified in the UK and in Nordic countries, including Denmark (n=16), Finland (n=2), and Sweden (n=4; figure 5b, appendix p 6).^10^ M1_UK_ isolates that possessed all 27 SNPs were, however, unique to the UK, except single sequences isolated in the USA in 2015^9^ and in Denmark in 2012,^10^ underlining the potential of the new lineage to disseminate internationally (figure 5B, appendix p 6).

## Discussion

The modern era of resurgent invasive *S pyogenes* infections was heralded by reports of virulent strains producing the scarlet fever toxin SpeA^17^ and subsequently dominated by the *emm*1 lineage.^10^ In this study, we showed that the originally polyclonal upsurge of scarlet fever in England has more recently been characterised by the emergence of a new *emm*1 *S pyogenes* lineage that produces significantly higher levels of SpeA than other contemporary *emm*1 strains. The new M1_UK_ lineage showed an apparent fitness advantage within the population, manifest during the scarlet fever seasons of 2015 and 2016. Phylogenetic analysis showed the emergent lineage to be the dominant cause of invasive *S pyogenes* infections in England in 2016, and indicated that isolates from symptomatic throat infections and scarlet fever represent the major reservoir for invasive infections. The data support the hypothesis that transmission of virulent *emm*1 strains with enhanced ability to cause scarlet fever could underlie the contemporaneous rise in invasive *S pyogenes* disease.

An unprecedented year-on-year increase in scarlet fever notifications, the underlying basis for which remains unclear,^3^, ^4^ has been seen in England since 2014. Among children aged 1–4 years, the incidence of scarlet fever in 2018 reached 1488 per 100 000.^18^ The increase in scarlet fever activity has followed secular changes in factors such as household structure (including use of childcare) and health-care use,^4^ as well as health-care policies,^19^ although causal links have not been established. One unforeseen consequence of medical practice change was that the capacity to investigate such an upsurge was undermined by a reduction in diagnostic testing at the national level. In northwest London, however, where strains are collected for epidemiological analysis, a significant increase in genotype *emm*4 pharyngitis strains was observed during the 2014 upsurge in scarlet fever, while *emm*3 was the main genotype associated with physician-reported scarlet fever.^4^ *emm*1 was infrequent in 2014, contrasting with the increase we observed between 2015 and 2016, when genotype *emm*1 *S pyogenes* became the dominant cause of upper respiratory tract infections regionally, and invasive disease notifications nationally.

Our genome sequence analysis revealed the emergence of a new *emm*1 lineage, separated from all other *emm*1 strains by 27 unique core SNPs, including three within a gene encoding a potential SpeA regulator, RofA. SpeA is usually the only phage-encoded superantigen in contemporary *emm*1 *S pyogenes*, and has been implicated in the re-emergence of severe invasive *S pyogenes* infections in the 1980s.^10^, ^17^ Although the roles of any specific SNPs were not investigated in the current study, we hypothesise that increased SpeA production by M1_UK_ strains might be an important contributor to the apparent fitness of the new lineage within the nasopharynx. As a phage-encoded superantigen, SpeA is hypothesised to trigger scarlet fever in susceptible children, and has been shown to permit nasopharyngeal infection in humanised models of murine streptococcal infection, plus potential induction of immunity when administered as a toxoid.^20^ SpeA can trigger B cell death and abrogate immunoglobulin secretion by the human tonsil, and might contribute to recurrent tonsillitis in children.^21^, ^22^ Thus, production of SpeA might augment the ability of *S pyogenes* to cause scarlet fever and paediatric pharyngitis. Whether population immunity to SpeA will lead to an eventual decline in the lineage will be of interest to monitor.

Griffiths type 1 *S pyogenes* strains^23^ (later designated serotype M1; genotype *emm*1) were the first to be classically associated with scarlet fever in the early 20th century. Although the oldest *emm*1 scarlet fever reference strains dating from the 1920s possess and produce phage-encoded SpeA^24^, *emm*1 strains circulating later in the mid-20th century lacked phage-encoded SpeA.^10^ The epidemic success of M1T1 clonal *emm*1 strains of *S pyogenes* that subsequently emerged in the 1980s is attributed to acquisition of a more active NADase–streptolysin O locus, as well as acquisition of phage-encoded *speA2,* an allele of *speA*, and the DNAse-encoding gene *sda*.^10^ It is, therefore, surprising that these epidemic *emm*1 strains, forming the M1_global_ lineage, apparently produce very little SpeA *in vitro*. The three SNPs identified in the *rofA* gene might contribute to the greater abundance of SpeA produced by the emergent M1_UK_ lineage strains, but appear insufficient alone to account for this change. Gene regulation could be affected by a number of the additional SNPs implicated. A shift to enhanced SpeA expression in the M1_UK_ lineage might represent just one component of a new phase in the evolution of *emm*1, although we have not yet explored other aspects of bacterial fitness.

Since the late 2000s, increased scarlet fever notifications have been reported in China,^25^ South Korea,^26^, ^27^ and Hong Kong.^28^ The M1_UK_ lineage is distinct from *emm*1 sequences identified in Asia, where different, non-*emm*1 genotypes have been reported as upsurge-associated, and incidence in very young children does not approach that reported in England.^25^ Although the acquisitions of novel prophages and antimicrobial resistance have been highlighted as selective pressures in China and Hong Kong,^28^ no evidence to suggest that these are factors in England, or that the emergence of a single lineage was a trigger for the original upsurge in England, has been reported.^3^

Sampling of the non-invasive reservoir of *S pyogenes* in our study was limited to a single region of England, and single seasons in 2014–16; thus, we cannot be certain about whether the non-invasive isolate *emm* genotype or M1_UK_ proportions altered outside these periods, and the analysis might be skewed through inadvertent inclusion of isolates from outbreaks. The collection used in this study, however, is the only systematic longitudinal collection of non-invasive strains available, and spans 2009–16. None of the samples included was identified as outbreak-associated, although clinical details were imperfect.

Groups of isolates differing by two SNPs or less were identified among non-invasive M1_UK_ isolates, suggesting recent transmission, whereas no such groups were observed among the M1_global_ non-invasive isolates, albeit these were from 2009–2014 when *emm*1 isolates were much less frequently identified. Whether the findings reflect inadvertent sampling of small outbreaks or greater outbreak potential cannot be addressed using these data.

Regardless of the drawbacks of regional sampling, the detection of a new lineage and increase in invasive *emm*1 infections prompted sequencing of invasive *emm*1 isolates referred to the national reference laboratory, which confirmed emergence of the same lineage at a national level, and excluded any artefact introduced by regional sampling. Our phylogenetic analysis of invasive isolates focused on the same season, in order to understand a rise in invasive disease, but published genome-sequenced invasive strains from the UK outside these months also showed emergence of the same lineage. Although the new lineage outcompeted the contemporary *emm*1 strains that were sequenced during the period studied, we do not know whether the lineage's success will endure.

Genetic analyses were limited by the nature of high-throughput short-read sequencing data, which do not provide fully assembled genome sequences for each strain and the phages therein; other lineage-specific differences might yet be identified using different approaches. Our study also did not address the mechanistic basis for fitness in the new lineage or the molecular basis for increased SpeA expression; links to specific SNPs are by association only, and detailed experimental work is underway to address such questions.

Scarlet fever is a visible and readily recognisable manifestation of *S pyogenes* infection that affects children aged 4–6 years.^3^, ^4^ Importantly, however, the surges observed in scarlet fever are accompanied by seasonal increases in streptococcal sore throat and tonsillitis in the wider population, also predominantly among children.^29^ Although we cannot rule out the possibility that the precursors (ie, intermediates) or founders of the M1_UK_ lineage were imported, we speculate that a generalised increase in *S pyogenes* activity in the wider population—which coincided with England's scarlet fever upsurges—might have provided the conditions required for adaptation and expansion of *emm*1 *S pyogenes*. Whether the M1_UK_ lineage will be suited to other environments is unknown; management of streptococcal sore throat differs greatly between countries,^30^ as do other important factors such as climate. We previously reported the emergence of a new *emm*89 lineage that had lost the capsule locus but gained an active NADase–streptolysin O locus, in addition to four other major recombination events.^31^ This *emm*89 lineage has now been identified across several continents.^32^ The identification of two members of the new M1_UK_ lineage among isolates outside the UK underlines the potential of such lineages to spread globally. Compared with other genotypes, *emm*1 *S pyogenes* has a recognised, heightened association with invasive infections.^9^ The expansion of such a lineage within the community reservoir of *S pyogenes* might be sufficient to explain England's recent increase in invasive infection. Further research to assess the likely effects of M1_UK_ on infection transmissibility, treatment response, disease burden, and severity is required, coupled with consideration of public health interventions to limit transmission where appropriate. Wider national and global surveillance will provide clearer understanding of the lineage's geographical reach and longer-term fitness, and permit enhanced public health readiness where necessary.
