# Supplementary material for: Emergence of dominant toxigenic M1T1 Streptococcus pyogenes clone during increased scarlet fever activity in England: a population-based molecular epidemiological study
Source: Lancet Infect Dis. 2019 Nov;19(11):1209–18. doi: 10.1016/S1473-3099(19)30446-3 (PMC6838661; doi:10.1016/S1473-3099(19)30446-3)
Supplement: Supplementary appendix [file mmc1.pdf]

THE LANCET
Infectious Diseases

Supplementary appendix

This appendix formed part of the original submission and has been peer reviewed. We post it as supplied by the authors.

Supplement to: Lynskey NN, Jauneikaite E, Li HK, et al. Emergence of dominant toxigenic M1T1 *Streptococcus pyogenes* clone during increased scarlet fever activity in England: a population-based molecular epidemiological study. *Lancet Infect Dis* 2019; published online Sept 10. http://dx.doi.org/10.1016/S1473-3099(19)30446-3.

SUPPLEMENTARY APPENDIX

Emergence of dominant toxigenic M1T1 *Streptococcus pyogenes* clone during increased scarlet fever activity in England: a population-based molecular epidemiological study

Nicola N Lynskey[1]*, Elita Jauneikaite[1,2,3]*, Ho Kwong Li[1], Xiangyun Zhi[1], Claire E Turner[4], Mia Mosavi[1,3], Max Pearson[1,3], Masanori Asai[1], Ludmila Lobkowicz[1], J. Yimmy Chow[3,5], Julian Parkhill[3,6], Theresa Lamagni[3,7] Victoria J Chalker[3,7], Shiranee Sriskandan[1,3]

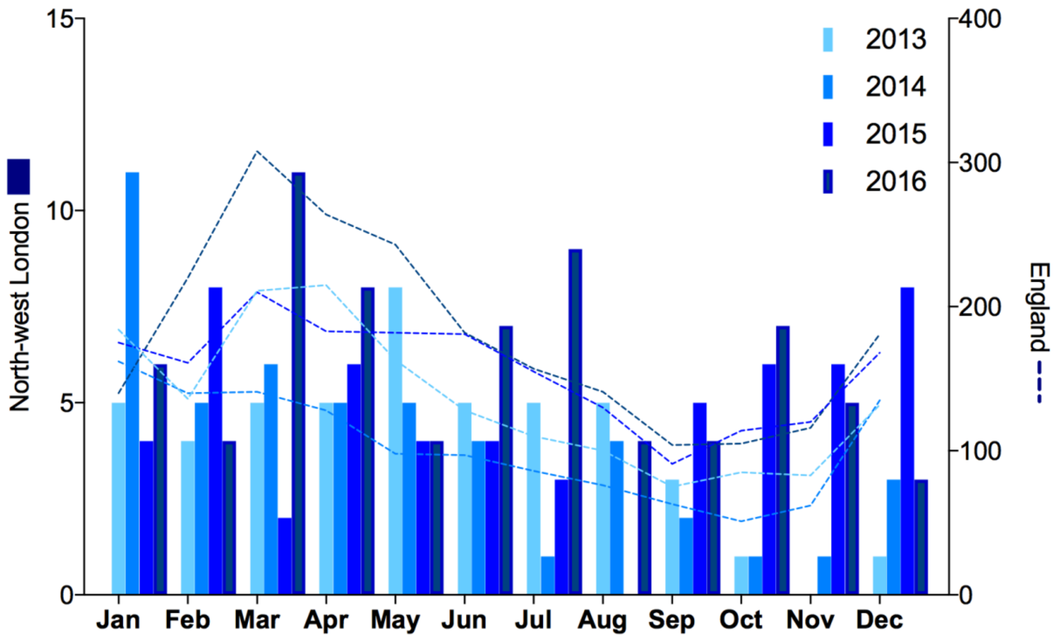

S1. Local and national invasive *S. pyogenes* infection notifications 2013-2016

Monthly numbers of notified cases of invasive *S. pyogenes* infections reported in northwest London (bars, left axis), and nationally (dashed lines, right axis). Surge of invasive *S. pyogenes* notifications in 2016 coincided with expansion of invasive isolates in $M1_{UK}$ lineage.

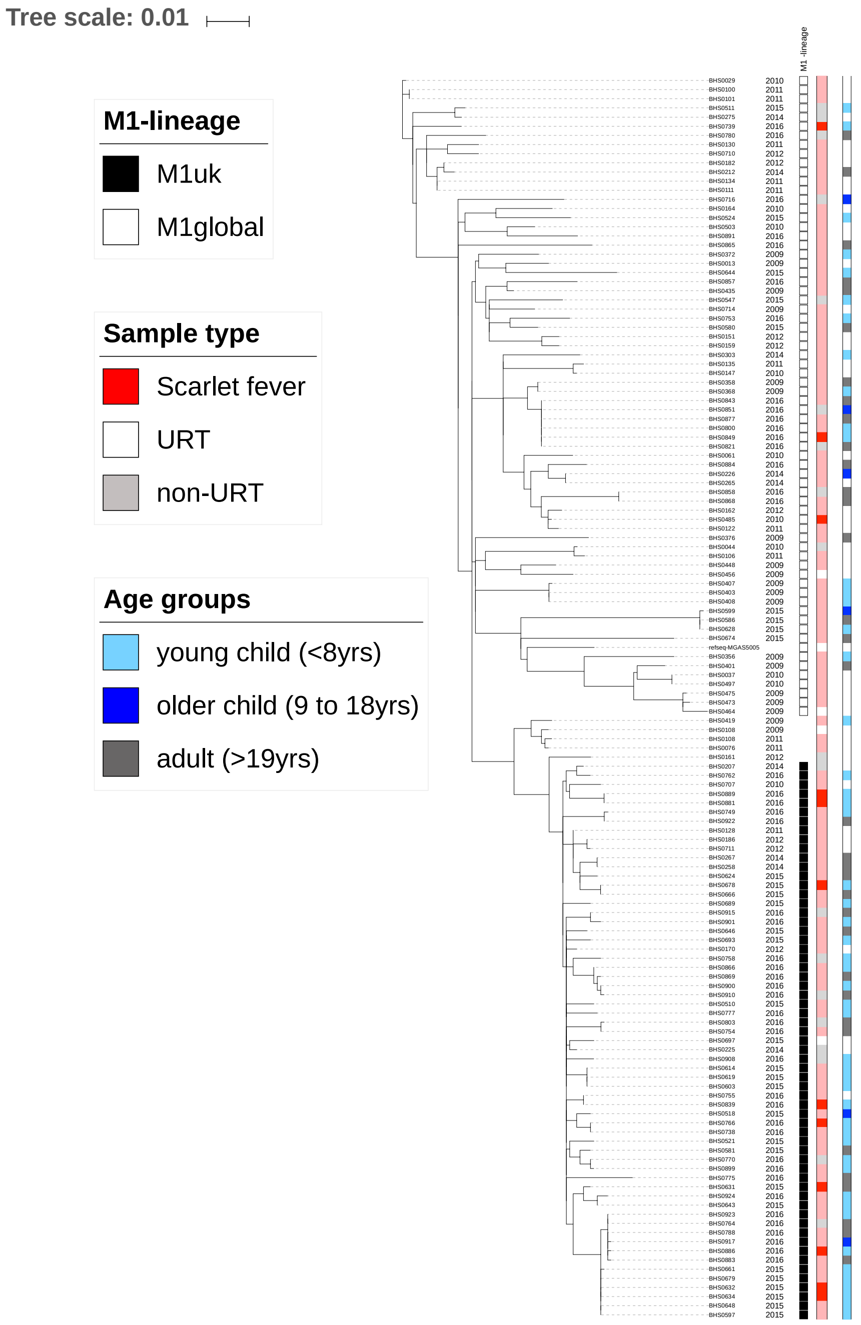

S2. Population structure of the non-invasive *emm*1 *S. pyogenes*.

Maximum likelihood rooted phylogenetic tree constructed from core SNPs (excluding prophage regions) of *emm*1 non-invasive isolates collected in North-West London between 2009-2016 (n=135). Year of isolation, M1 lineage, non-invasive sample type, mention of scarlet fever, and age group are indicated in individual columns; gaps indicate no clinical information was available. An empty square without borders in the M1 lineage column indicates intermediate isolates (n=5) with 13 or 23SNPs (including 3*rofA* mutations) of the 27SNPs that define the $M1_{UK}$ lineage; none of the M1global strains had these 3 *rofA* mutations. Scale bar indicates nucleotide substitutions per site.

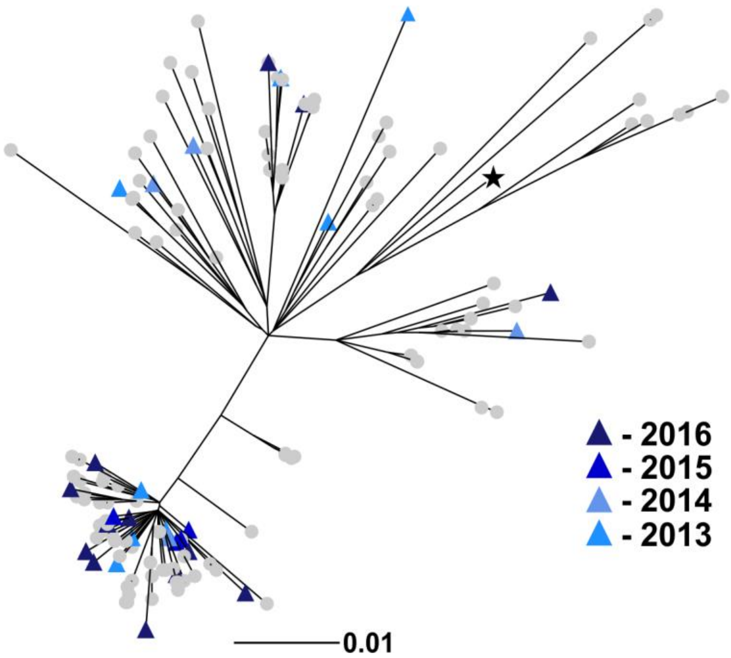

S3. Phylogenetic relationship between invasive and non-invasive *emm*1 *S. pyogenes* isolates in northwest London.

Maximum likelihood phylogenetic tree constructed from core SNPs (excluding prophage regions) comparing *emm*1 non-invasive isolates grey circles collected in northwest London in 2009-2016 (n=135 Jan-Dec 2009-2013; March-May 2014-2016) with contemporaneous invasive isolates from the same region, blue triangles, (n=31, March-May 2013-2016), shading according to year. Strains from the $M1_{UK}$ emergent lineage were responsible for both non-invasive and invasive infections with some indistinguishable strains. Star; reference strain MGAS5005. Scale bar represents nucleotide substitutions per site.

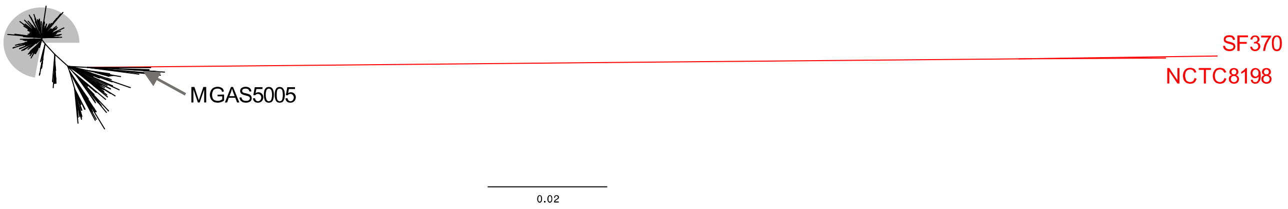

S4 Population structure of *emm*1 isolates from UK in comparison to historical scarlet fever reference strains SF370 and NCTC8198.

Maximum likelihood unrooted phylogenetic tree constructed from core SNPs (excluding prophage regions) comparing UK sterile site *S. pyogenes emm*1 sequences from 2013-2016 (n=552) and non-invasive North-West London *S. pyogenes emm*1 sequences 2009-2016 (n=135) with scarlet fever isolates SF370 (AE004092) (SpeC-positive) and NCTC8198 (LN831034)(SpeA-positive). Scale bar indicates nucleotide substitutions per site.

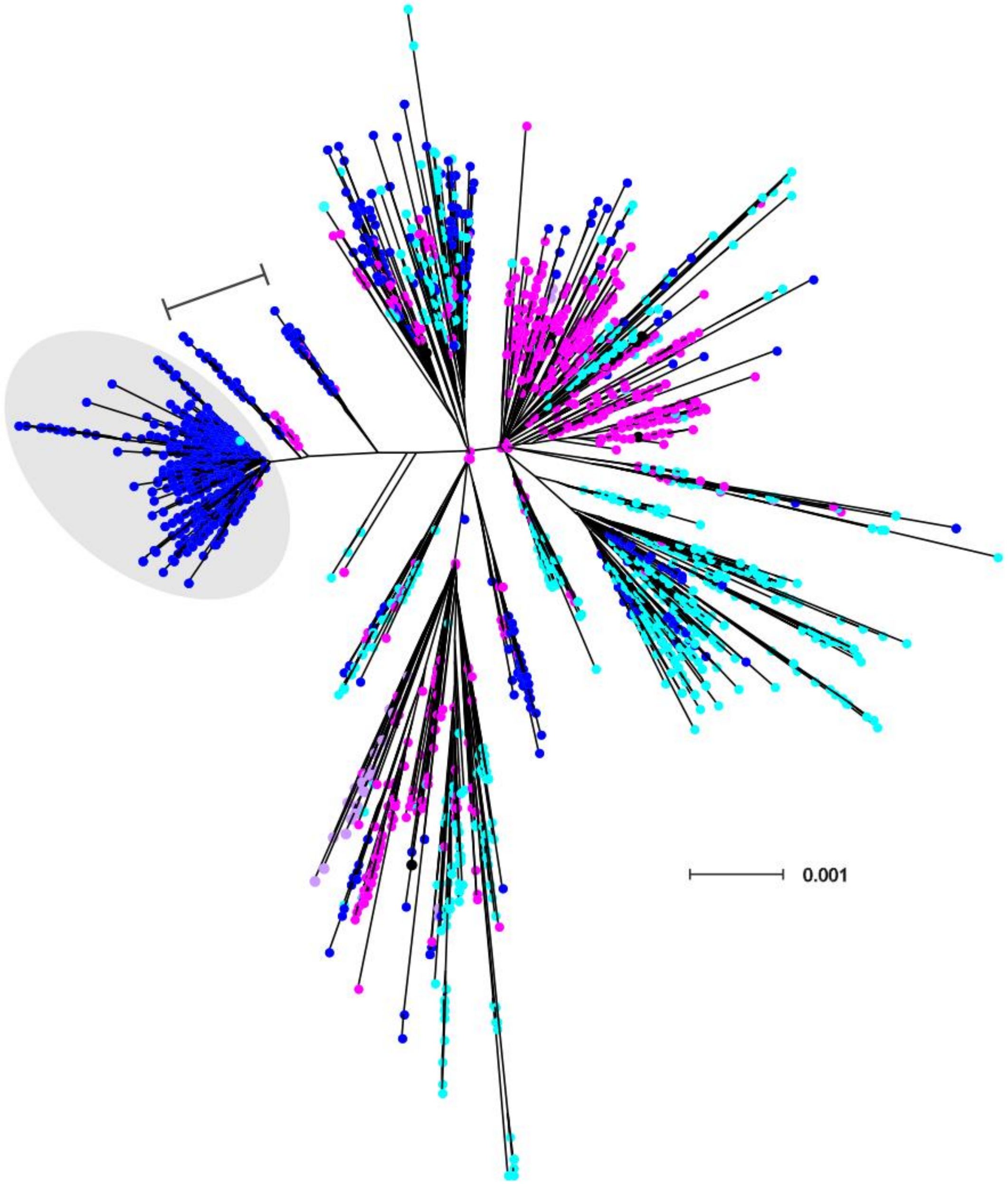

S5. Emergent $M1_{UK}$ lineage in a global context.

Maximum likelihood unrooted phylogenetic tree constructed from core SNPs (excluding prophage regions) comparing all sequenced UK *emm*1 isolates (blue) with the global *emm*1 population: North America (turquoise), Nordic countries (pink), and Asia (lilac) as shown in Figure 5B (n=2800 isolates). Shading in grey indicates the emergent lineage $M1_{UK}$. While UK and international *emm*1 isolates arise throughout the tree, isolates within the $M1_{UK}$ lineage are exclusively from UK, bar two single isolates from Denmark and USA (brought forwards for clarity). Straight line marker indicates branches representing intermediate isolates with 13-23SNPs (including 3 *rofA* mutations) of the 27SNPs identified in $M1_{UK}$ lineage strains. Other isolates did not have the 3 specific *rofA* mutations identified. Scale bar indicates nucleotide substitutions per site.

Supplementary table 1: $M1_{UK}$ Lineage-associated SNPs

Position in MGAS5005	Gene locus	Gene	Product	S/NS	Ref	SNP
115646	M5005_Spy0106	rofA	Transcriptional regulator	NS	C	T
116162	M5005_Spy0106	rofA	Transcriptional regulator	NS	A	C
116163	M5005_Spy0106	rofA	Transcriptional regulator	NS	C	A
250832	M5005_Spy0243		ABC transporter-associated protein	S	T	C
513254	M5005_Spy0525		galactose-6-phosphate isomerase LacB subunit	NS	G	T
528360	Intergenic		-	-	A	T
563631	M5005_Spy0566	sagE	streptolysin S putative self-immunity protein	NS	G	A
613633	M5005_Spy0609		phosphoglycerol transferase	NS	T	C
626494	M5005_Spy0623		methyltransferase	S	G	A
661707	M5005_Spy0656	trmD	tRNA (guanine-N(1)-)-methyltransferase	NS	G	A
730823	M5005_Spy0727	recJ	single-stranded-DNA-specific exonuclease	NS	C	T
784467	M5005_Spy0779		putative membrane spanning protein	S	T	C
819098	M5005_Spy0825	murB	UDP-N-acetylenolpyruvoylglucosamine reductase	NS	G	A
923079	M5005_Spy0933		putative NADH-dependent flavin oxidoreductase	NS	G	A
942633	M5005_Spy0951	pstB	phosphate transport ATP-binding protein	NS	G	T
983438	Intergenic		-	-	G	C
1082253	M5005_Spy1108	metK2	S-adenosylmethionine synthetase	NS	C	T
1238124	M5005_Spy1282	msrA	peptide methionine sulfoxide reductase	NS	G	A
1238673	M5005_Spy1283	tlpA	thiol:disulfide interchange protein	NS	G	A
1251193	M5005_Spy1293		hypothetical protein	NS	G	A
1373176	M5005_Spy1400		PTS system, galactose-specific IIB component	NS	C	A
1407497	M5005_Spy1439		portal protein	NS	C	T
1446116	M5005_Spy1490		3-oxoacyl-[acyl-carrier protein] reductase	S	C	T
1535209	Intergenic		-	-	A	G
1702540	M5005_Spy1714	gldA	glycerol dehydrogenase	STOP	C	T
1734749	M5005_Spy1772		glutamate formimidoyltransferase	NS	G	A
1828734	M5005_Spy1860		putative membrane spanning protein	NS	G	A

S denotes synonymous mutation; NS denotes non-synonymous mutation; STOP denotes a nonsense mutation; Reference strain used is MGAS5005

Supplementary table 2: Non-invasive *S. pyogenes emm*1 strains isolated in North-West London 2009-2016 that underwent whole genome sequencing as part of this study (n=135)

ENA	Strain	Lineage	Year
**ERS1020045**	**BHS0013**	**$M1_{global}$**	**2009***
ERS1020076	BHS0029	$M1_{global}$	2010*
**ERS1020092**	**BHS0037**	**$M1_{global}$**	**2010***
ERS1020107	BHS0044	$M1_{global}$	2010
ERS1020141	BHS0061	$M1_{global}$	2010*
ERS1020171	BHS0076	$M1_{inter}$	2011*
ERS1020034	BHS0100	$M1_{global}$	2011*
ERS1020036	BHS0101	$M1_{global}$	2011*
ERS1020046	BHS0106	$M1_{global}$	2011*
**ERS1020050**	**BHS0108**	**$M1_{inter}$**	**2011***
ERS1020056	BHS0111	$M1_{global}$	2011*
ERS1020079	BHS0122	$M1_{global}$	2011*
**ERS1020090**	**BHS0128**	**$M1_{UK}$**	**2011***
**ERS1020095**	**BHS0130**	**$M1_{global}$**	**2011***
ERS1020102	BHS0134	$M1_{global}$	2011*
**ERS1020104**	**BHS0135**	**$M1_{global}$**	**2011***
ERS1020128	BHS0147	$M1_{global}$	2010*
**ERS1020136**	**BHS0151**	**$M1_{global}$**	**2012***
ERS1020152	BHS0159	$M1_{global}$	2012*
ERS1020156	BHS0161	$M1_{inter}$	2012
**ERS1020158**	**BHS0162**	**$M1_{global}$**	**2012***
ERS1020162	BHS0164	$M1_{global}$	2010*
**ERS1020174**	**BHS0170**	**$M1_{UK}$**	**2012***
ERS1020199	BHS0182	$M1_{global}$	2012*
ERS1020207	BHS0186	$M1_{UK}$	2012*
ERS1020240	BHS0207	$M1_{UK}$	2014
ERS1020250	BHS0212	$M1_{global}$	2014*
ERS1020275	BHS0225	$M1_{UK}$	2014
ERS1020277	BHS0226	$M1_{global}$	2014*
**ERS1020341**	**BHS0258**	**$M1_{UK}$**	**2014***
ERS1020354	BHS0265	$M1_{global}$	2014*
ERS1020358	BHS0267	$M1_{UK}$	2014*

ERS1020374	BHS0275	$M1_{global}$	2014
**ERS1020253**	**BHS0303**	**$M1_{global}$**	**2014***
ERS1020361	BHS0356	$M1_{global}$	2009*
ERS1020365	BHS0358	$M1_{global}$	2009*
ERS1020385	BHS0368	$M1_{global}$	2009*
ERS1020393	BHS0372	$M1_{global}$	2009*
ERS1020398	BHS0376	$M1_{global}$	2009*
ERS1020429	BHS0401	$M1_{global}$	2009*
ERS1020433	BHS0403	$M1_{global}$	2009*
ERS1020441	BHS0407	$M1_{global}$	2009*
ERS1020443	BHS0408	$M1_{global}$	2009*
ERS1020465	BHS0419	$M1_{inter}$	2009*
ERS1020497	BHS0435	$M1_{global}$	2009*
ERS1020523	BHS0448	$M1_{global}$	2009*
ERS1020539	BHS0456	$M1_{global}$	2009
ERS1020551	BHS0462	$M1_{inter}$	2009
ERS1020555	BHS0464	$M1_{global}$	2009
ERS1020572	BHS0473	$M1_{global}$	**2009***
ERS1020576	BHS0475	$M1_{global}$	2009*
ERS1020435	BHS0485	$M1_{global}$	2010*
**ERS1020460**	**BHS0497**	**$M1_{global}$**	**2010***
ERS1020472	BHS0503	$M1_{global}$	2010*
**ERS1020486**	**BHS0510**	**$M1_{UK}$**	**2015***
ERS1020488	BHS0511	$M1_{global}$	2015
ERS1020502	BHS0518	$M1_{UK}$	2015*
**ERS1020508**	**BHS0521**	**$M1_{UK}$**	**2015***
**ERS1020514**	**BHS0524**	**$M1_{global}$**	**2015***
ERS1020561	BHS0547	$M1_{global}$	2015
ERS1020602	BHS0580	$M1_{global}$	2015*
**ERS1020603**	**BHS0581**	**$M1_{UK}$**	**2015***
ERS1020613	BHS0586	$M1_{global}$	2015*
ERS1020634	BHS0597	$M1_{UK}$	2015*
ERS1020638	BHS0599	$M1_{global}$	2015*
ERS1020646	BHS0603	$M1_{UK}$	2015*
ERS1020668	BHS0614	$M1_{UK}$	2015*

ERS1020679	BHS0619	$M1_{UK}$	2015*
ERS1020688	BHS0624	$M1_{UK}$	2015*
ERS1020696	BHS0628	$M1_{global}$	2015*
**ERS1020702**	**BHS0631**	$\mathbf{M1_{UK}}$	**2015***
ERS1020703	BHS0632	$M1_{UK}$	2015*
ERS1020705	BHS0634	$M1_{UK}$	2015*
**ERS1020714**	**BHS0643**	$\mathbf{M1_{UK}}$	**2015***
ERS1020715	BHS0644	$M1_{global}$	2015*
ERS1020717	BHS0646	$M1_{UK}$	2015*
ERS1020719	BHS0648	$M1_{UK}$	2015*
ERS1020732	BHS0661	$M1_{UK}$	2015*
ERS1020604	BHS0666	$M1_{UK}$	2015*
**ERS1020620**	**BHS0674**	$\mathbf{M1_{global}}$	**2015***
ERS1020629	BHS0678	$M1_{UK}$	2015*
ERS1020631	BHS0679	$M1_{UK}$	2015*
ERS1020651	BHS0689	$M1_{UK}$	2015*
ERS1020659	BHS0693	$M1_{UK}$	2015*
ERS1020667	BHS0697	$M1_{UK}$	2015
ERS1020687	BHS0707	$M1_{UK}$	2010*
ERS1020692	BHS0710	$M1_{global}$	2012*
**ERS1020694**	**BHS0711**	$\mathbf{M1_{UK}}$	**2012***
ERS1020701	BHS0714	$M1_{global}$	**2009***
ERS1463073	BHS0716	$M1_{global}$	2016
ERS1463078	BHS0738	$M1_{UK}$	2016*
ERS1463079	BHS0739	$M1_{global}$	2016*
ERS1463083	BHS0749	$M1_{UK}$	2016*
ERS1463084	BHS0753	$M1_{global}$	2016*
ERS1463085	BHS0754	$M1_{UK}$	2016*
ERS1463086	BHS0755	$M1_{UK}$	2016*
ERS1463087	BHS0758	$M1_{UK}$	2016
ERS1463088	BHS0762	$M1_{UK}$	2016*
ERS1463089	BHS0764	$M1_{UK}$	2016
ERS1463090	BHS0766	$M1_{UK}$	2016*
ERS1463091	BHS0770	$M1_{UK}$	2016
ERS1463092	BHS0775	$M1_{UK}$	2016*

ERS1463093	BHS0777	$M1_{UK}$	2016*
ERS1463095	BHS0780	$M1_{global}$	2016
ERS1463096	BHS0788	$M1_{UK}$	2016*
ERS1463100	BHS0800	$M1_{global}$	2016*
ERS1463101	BHS0803	$M1_{UK}$	2016
ERS1463102	BHS0821	$M1_{global}$	2016
ERS1463104	BHS0839	$M1_{UK}$	2016*
ERS1463105	BHS0843	$M1_{global}$	2016*
ERS1463106	BHS0849	$M1_{global}$	2016*
ERS1463107	BHS0851	$M1_{global}$	2016
ERS1463108	BHS0857	$M1_{global}$	2016*
ERS1463109	BHS0858	$M1_{global}$	2016
ERS1463110	BHS0865	$M1_{global}$	2016*
ERS1463111	BHS0866	$M1_{UK}$	2016*
ERS1463112	BHS0868	$M1_{global}$	2016*
ERS1463113	BHS0869	$M1_{UK}$	2016*
ERS1463114	BHS0877	$M1_{global}$	2016*
ERS1463115	BHS0881	$M1_{UK}$	2016*
ERS1463116	BHS0883	$M1_{UK}$	2016*
ERS1463117	BHS0884	$M1_{global}$	2016*
ERS1463118	BHS0886	$M1_{UK}$	2016*
ERS1463119	BHS0889	$M1_{UK}$	2016*
ERS1463120	BHS0891	$M1_{global}$	2016*
ERS1463121	BHS0899	$M1_{UK}$	2016*
ERS1463122	BHS0900	$M1_{UK}$	2016*
ERS1463123	BHS0901	$M1_{UK}$	2016*
ERS1463124	BHs0908	$M1_{UK}$	2016
ERS1463125	BHS0910	$M1_{UK}$	2016
ERS1463126	BHS0915	$M1_{UK}$	2016
ERS1463127	BHS0917	$M1_{UK}$	2016*
ERS1463129	BHS0922	$M1_{UK}$	2016*
ERS1463130	BHS0923	$M1_{UK}$	2016*
ERS1463131	BHS0924	$M1_{UK}$	2016*

Grey shading with bold font denotes isolates used for SpeA expression analysis;
$M1_{inter}$ indicates intermediate isolate
* Upper respiratory tract (URT) strains. 2014-2016 isolates were collected for sequencing March-May

Supplementary table 3: *Emm1 S. pyogenes* isolated from invasive disease cases England and Wales March-May of 2013 and 2016 that underwent whole genome sequencing as part of this study (n=446; of which 425 were sterile site)

ENA	Isolate	Sterile site (yes/no)	Year
ERS1594710	PHEGAS001	Yes	2013
ERS1594711	PHEGAS002	Yes	2013
ERS1594712	PHEGAS003	Yes	2013
ERS1594713	PHEGAS004	Yes	2013
ERS1594714	PHEGAS005	Yes	2013
ERS1594715	PHEGAS006	Yes	2013
ERS1594716	PHEGAS007	Yes	2013
ERS1594717	PHEGAS008	No	2013
ERS1594718	PHEGAS009	Yes	2013
ERS1594719	PHEGAS010	Yes	2013
ERS1594720	PHEGAS011	Yes	2013
ERS1594721	PHEGAS012	Yes	2013
ERS1594722	PHEGAS013	Yes	2013
ERS1594723	PHEGAS014	Yes	2013
ERS1594724	PHEGAS015	Yes	2013
ERS1594725	PHEGAS016	Yes	2013
ERS1594726	PHEGAS017	Yes	2013
ERS1594727	PHEGAS018	Yes	2013
ERS1594728	PHEGAS019	Yes	2013
ERS1594729	PHEGAS020	Yes	2013[W]
ERS1594730	PHEGAS021	Yes	2013
ERS1594731	PHEGAS022	Yes	2013[L]
ERS1594732	PHEGAS023	Yes	2013
ERS1594733	PHEGAS024	Yes	2013
ERS1594734	PHEGAS025	Yes	2013
ERS1594735	PHEGAS026	Yes	2013[L]
ERS1594736	PHEGAS027	Yes	2013
ERS1594737	PHEGAS028	Yes	2013
ERS1594738	PHEGAS029	Yes	2013
ERS1594739	PHEGAS030	Yes	2013
ERS1594740	PHEGAS031	Yes	2013
ERS1594741	PHEGAS032	Yes	2013
ERS1594742	PHEGAS033	Yes	2013
ERS1594743	PHEGAS034	Yes	2013
ERS1594744	PHEGAS035	Yes	2013

ERS1594745	PHEGAS036	Yes	2013
ERS1594746	PHEGAS037	Yes	2013
ERS1594747	PHEGAS038	Yes	2013
ERS1594748	PHEGAS039	Yes	2013
ERS1594749	PHEGAS040	Yes	2013
ERS1594750	PHEGAS041	Yes	2013
ERS1594751	PHEGAS042	Yes	2013[W]
ERS1594752	PHEGAS043	Yes	2013
ERS1594753	PHEGAS044	Yes	2013
ERS1594754	PHEGAS045	Yes	2013
ERS1594755	PHEGAS046	Yes	2013
ERS1594756	PHEGAS047	Yes	2013
ERS1594757	PHEGAS048	Yes	2013
ERS1594758	PHEGAS049	Yes	2013
ERS1594759	PHEGAS050	Yes	2013[L]
ERS1594760	PHEGAS051	Yes	2013
ERS1594762	PHEGAS053	Yes	2013
ERS1594763	PHEGAS054	Yes	2013
ERS1594764	PHEGAS055	Yes	2013
ERS1594765	PHEGAS056	Yes	2013
ERS1594766	PHEGAS057	Yes	2013
ERS1594767	PHEGAS058	Yes	2013
ERS1594768	PHEGAS059	Yes	2013
ERS1594769	PHEGAS060	Yes	2013
ERS1594770	PHEGAS061	Yes	2013
ERS1594771	PHEGAS062	Yes	2013
ERS1594772	PHEGAS063	Yes	2013
ERS1594773	PHEGAS064	No	2013
ERS1594774	PHEGAS065	No	2013
ERS1594775	PHEGAS066	Yes	2013
ERS1594776	PHEGAS067	Yes	2013[W]
ERS1594777	PHEGAS068	Yes	2013
ERS1594778	PHEGAS069	Yes	2013
ERS1594779	PHEGAS070	Yes	2013
ERS1594780	PHEGAS071	Yes	2013
ERS1594781	PHEGAS072	Yes	2013
ERS1594782	PHEGAS073	Yes	2013
ERS1594783	PHEGAS074	Yes	2013
ERS1594784	PHEGAS075	Yes	2013

ERS1594785	PHEGAS076	Yes	2013
ERS1594786	PHEGAS077	Yes	2013
ERS1594787	PHEGAS078	Yes	2013[W]
ERS1594788	PHEGAS079	No	2013
ERS1594805	PHEGAS080	Yes	2013
ERS1594807	PHEGAS082	Yes	2013
ERS1594808	PHEGAS083	Yes	2013[L]
ERS1594809	PHEGAS084	Yes	2013
ERS1594810	PHEGAS085	Yes	2013
ERS1594811	PHEGAS086	No	2013
ERS1594812	PHEGAS087	Yes	2013
ERS1594813	PHEGAS088	Yes	2013
ERS1594814	PHEGAS089	Yes	2013
ERS1594815	PHEGAS090	Yes	2013
ERS1594816	PHEGAS091	Yes	2013
ERS1594817	PHEGAS092	Yes	2013
ERS1594818	PHEGAS093	Yes	2013
ERS1594819	PHEGAS094	Yes	2013
ERS1594821	PHEGAS096	Yes	2013
ERS1594822	PHEGAS097	Yes	2013
ERS1594823	PHEGAS098	Yes	2013
ERS1594824	PHEGAS099	Yes	2013
ERS1594825	PHEGAS100	Yes	2013
ERS1594826	PHEGAS101	Yes	2013
ERS1594827	PHEGAS102	Yes	2013
ERS1594828	PHEGAS103	Yes	2013
ERS1594829	PHEGAS104	Yes	2013
ERS1594830	PHEGAS105	Yes	2013[L]
ERS1594831	PHEGAS106	Yes	2013
ERS1594832	PHEGAS107	No	2013
ERS1594833	PHEGAS108	Yes	2013
ERS1594834	PHEGAS109	No	2013
ERS1594835	PHEGAS110	Yes	2013
ERS1594836	PHEGAS111	Yes	2013
ERS1594837	PHEGAS112	Yes	2013
ERS1594838	PHEGAS113	Yes	2013
ERS1594839	PHEGAS114	Yes	2013
ERS1594840	PHEGAS115	Yes	2013
ERS1594841	PHEGAS116	Yes	2013

ERS1594842	PHEGAS117	Yes	2013
ERS1594843	PHEGAS118	Yes	2013
ERS1594844	PHEGAS119	Yes	2013
ERS1594845	PHEGAS120	Yes	2013
ERS1594846	PHEGAS121	Yes	2013
ERS1594847	PHEGAS122	Yes	2013
ERS1594848	PHEGAS123	Yes	2013
ERS1594849	PHEGAS124	Yes	2013
ERS1594850	PHEGAS125	Yes	2013
ERS1594851	PHEGAS126	Yes	2013
ERS1594852	PHEGAS127	Yes	2013
ERS1594853	PHEGAS128	Yes	2013
ERS1594854	PHEGAS129	Yes	2013
ERS1594855	PHEGAS130	Yes	2013
ERS1594858	PHEGAS131	Yes	2013
ERS1594859	PHEGAS132	Yes	2013
ERS1594860	PHEGAS133	Yes	2013
ERS1594861	PHEGAS134	Yes	2013
ERS1594862	PHEGAS135	No	2013
ERS1594863	PHEGAS136	Yes	2013
ERS1594864	PHEGAS137	Yes	2013
ERS1594856	PHEGAS138	Yes	2013
ERS1594865	PHEGAS140	Yes	2013
ERS1594866	PHEGAS141	Yes	2013[W]
ERS1594867	PHEGAS142	Yes	2013
ERS1594868	PHEGAS143	Yes	2013
ERS1594869	PHEGAS144	Yes	2013
ERS1594870	PHEGAS145	Yes	2013
ERS1594871	PHEGAS146	Yes	2013
ERS1594872	PHEGAS147	Yes	2013
ERS1594873	PHEGAS148	Yes	2013
ERS1594874	PHEGAS149	Yes	2013
ERS1594875	PHEGAS150	Yes	2013
ERS1594876	PHEGAS151	Yes	2013
ERS1594877	PHEGAS152	Yes	2013
ERS1594878	PHEGAS153	Yes	2013
ERS1594879	PHEGAS154	No	2013
ERS1594880	PHEGAS155	No	2013
ERS1594881	PHEGAS156	Yes	2013

ERS1594882	PHEGAS157	Yes	2013
ERS1594883	PHEGAS158	Yes	2013
ERS1594789	PHEGAS159	Yes	2013
ERS1594790	PHEGAS160	Yes	2013[L]
ERS1594791	PHEGAS161	Yes	2013
ERS1594792	PHEGAS162	Yes	2013
ERS1594793	PHEGAS163	Yes	2013
ERS1594794	PHEGAS164	Yes	2013
ERS1594795	PHEGAS165	Yes	2013[L]
ERS1594796	PHEGAS166	Yes	2013
ERS1594797	PHEGAS167	Yes	2013
ERS1594798	PHEGAS168	Yes	2013
ERS1594799	PHEGAS169	Yes	2013
ERS1594800	PHEGAS170	No	2013
ERS1594801	PHEGAS171	Yes	2013
ERS1594802	PHEGAS172	Yes	2013
ERS1594803	PHEGAS173	Yes	2013[W]
ERS1594804	PHEGAS174	Yes	2013
ERS1594884	PHEGAS175	Yes	2013
ERS1594885	PHEGAS176	Yes	2013
ERS1594886	PHEGAS177	Yes	2013
ERS1594888	PHEGAS178	Yes	2013
ERS1594889	PHEGAS179	Yes	2013
ERS1594890	PHEGAS180	Yes	2013
ERS1594891	PHEGAS181	Yes	2013
ERS1594892	PHEGAS182	Yes	2013
ERS1594893	PHEGAS183	Yes	2013
ERS1594894	PHEGAS184	Yes	2013
ERS1594887	PHEGAS185	Yes	2013
ERS1594895	PHEGAS186	Yes	2013
ERS1594896	PHEGAS187	Yes	2013
ERS1594897	PHEGAS188	Yes	2013
ERS1594898	PHEGAS189	Yes	2013
ERS1594899	PHEGAS190	Yes	2013
ERS1595155	PHEGAS196	Yes	2013[L]
ERS1595156	PHEGAS197	Yes	2016
ERS1595158	PHEGAS198	Yes	2016[L]
ERS1595162	PHEGAS200	Yes	2016
ERS1595164	PHEGAS201	Yes	2016

ERS1595166	PHEGAS202	No	2016
ERS1595167	PHEGAS203	Yes	2016
ERS1595169	PHEGAS204	Yes	2016
ERS1595172	PHEGAS206	Yes	2016[L]
ERS1594979	PHEGAS207	Yes	2016
ERS1594980	PHEGAS208	Yes	2016
ERS1594981	PHEGAS209	Yes	2016
ERS1594982	PHEGAS210	Yes	2016
ERS1594983	PHEGAS211	Yes	2016
ERS1594984	PHEGAS212	Yes	2016
ERS1594985	PHEGAS213	Yes	2016
ERS1594986	PHEGAS214	Yes	2016
ERS1594987	PHEGAS215	Yes	2016
ERS1594988	PHEGAS216	Yes	2016
ERS1594989	PHEGAS217	Yes	2016
ERS1594990	PHEGAS218	Yes	2016
ERS1594991	PHEGAS219	No	2016
ERS1594992	PHEGAS220	Yes	2016
ERS1594993	PHEGAS221	Yes	2016
ERS1594994	PHEGAS222	Yes	2016
ERS1595116	PHEGAS223	Yes	2016
ERS1595118	PHEGAS224	Yes	2016
ERS1595119	PHEGAS225	Yes	2016
ERS1595121	PHEGAS226	Yes	2016
ERS1595123	PHEGAS227	Yes	2016
ERS1595124	PHEGAS228	Yes	2016
ERS1595126	PHEGAS229	Yes	2016
ERS1595128	PHEGAS230	Yes	2016
ERS1595129	PHEGAS231	Yes	2016
ERS1595131	PHEGAS232	Yes	2016
ERS1595133	PHEGAS233	Yes	2016
ERS1595134	PHEGAS234	Yes	2016
ERS1595136	PHEGAS235	Yes	2016
ERS1595138	PHEGAS236	Yes	2016
ERS1595139	PHEGAS237	Yes	2016
ERS1594900	PHEGAS238	Yes	2016
ERS1594901	PHEGAS239	Yes	2016
ERS1594902	PHEGAS240	Yes	2016
ERS1594903	PHEGAS241	Yes	2016

ERS1594904	PHEGAS242	Yes	2016
ERS1594905	PHEGAS243	Yes	2016
ERS1594906	PHEGAS244	Yes	2016
ERS1594907	PHEGAS245	Yes	2016
ERS1594908	PHEGAS246	No	2016
ERS1594909	PHEGAS247	Yes	2016
ERS1594910	PHEGAS248	Yes	2016[W]
ERS1594911	PHEGAS249	Yes	2013
ERS1594912	PHEGAS250	Yes	2016
ERS1594913	PHEGAS251	Yes	2016
ERS1594914	PHEGAS252	Yes	2016
ERS1594915	PHEGAS253	Yes	2016
ERS1594916	PHEGAS254	Yes	2016
ERS1594917	PHEGAS255	Yes	2016
ERS1594918	PHEGAS256	Yes	2016
ERS1594919	PHEGAS257	No	2016
ERS1594920	PHEGAS258	No	2016
ERS1594921	PHEGAS259	Yes	2016
ERS1594922	PHEGAS260	Yes	2013
ERS1594923	PHEGAS261	Yes	2016
ERS1594924	PHEGAS262	Yes	2016
ERS1594925	PHEGAS263	Yes	2016
ERS1594926	PHEGAS264	Yes	2013
ERS1594927	PHEGAS265	Yes	2016
ERS1594928	PHEGAS266	Yes	2016
ERS1594929	PHEGAS267	Yes	2016
ERS1594930	PHEGAS268	Yes	2016
ERS1594931	PHEGAS269	Yes	2016
ERS1594932	PHEGAS270	Yes	2016
ERS1594933	PHEGAS271	Yes	2016
ERS1594934	PHEGAS272	Yes	2016
ERS1594935	PHEGAS273	Yes	2016
ERS1594936	PHEGAS274	Yes	2016
ERS1594937	PHEGAS275	Yes	2016
ERS1594938	PHEGAS276	Yes	2016
ERS1594939	PHEGAS277	Yes	2016
ERS1594940	PHEGAS278	Yes	2016
ERS1594941	PHEGAS279	Yes	2016
ERS1594942	PHEGAS280	Yes	2016

ERS1594943	PHEGAS281	Yes	2016
ERS1594944	PHEGAS282	Yes	2016
ERS1594945	PHEGAS283	Yes	2016
ERS1594946	PHEGAS284	Yes	2016
ERS1594947	PHEGAS285	Yes	2016
ERS1594948	PHEGAS286	Yes	2016
ERS1594949	PHEGAS287	Yes	2016
ERS1594950	PHEGAS288	Yes	2016
ERS1594951	PHEGAS289	Yes	2016
ERS1594952	PHEGAS290	Yes	2016
ERS1594953	PHEGAS291	No	2016
ERS1594954	PHEGAS292	Yes	2016
ERS1594955	PHEGAS293	Yes	2016
ERS1594956	PHEGAS294	Yes	2016
ERS1594957	PHEGAS295	Yes	2016
ERS1594958	PHEGAS296	Yes	2016
ERS1594959	PHEGAS297	Yes	2016
ERS1594960	PHEGAS298	Yes	2016
ERS1594961	PHEGAS299	Yes	2016
ERS1594962	PHEGAS300	Yes	2016
ERS1594963	PHEGAS301	Yes	2016 [L]
ERS1594964	PHEGAS302	Yes	2016
ERS1594965	PHEGAS303	Yes	2016
ERS1594966	PHEGAS304	Yes	2016
ERS1594967	PHEGAS305	Yes	2016
ERS1594968	PHEGAS306	Yes	2016
ERS1594969	PHEGAS307	Yes	2016
ERS1594970	PHEGAS308	Yes	2016
ERS1594971	PHEGAS309	Yes	2016
ERS1594972	PHEGAS310	Yes	2016
ERS1594973	PHEGAS311	Yes	2016
ERS1594974	PHEGAS312	Yes	2016
ERS1594975	PHEGAS313	Yes	2016[L]
ERS1594976	PHEGAS314	Yes	2016
ERS1594977	PHEGAS315	Yes	2016
ERS1594978	PHEGAS316	Yes	2016
ERS1595187	PHEGAS317	Yes	2016[L]
ERS1595189	PHEGAS318	Yes	2016
ERS1595191	PHEGAS319	Yes	2016

ERS1595176	PHEGAS320	Yes	2016
ERS1595193	PHEGAS321	Yes	2016
ERS1595195	PHEGAS322	Yes	2016
ERS1595196	PHEGAS323	Yes	2016
ERS1595200	PHEGAS325	Yes	2016
ERS1595201	PHEGAS326	Yes	2016
ERS1595203	PHEGAS327	Yes	2016
ERS1595184	PHEGAS328	Yes	2016
ERS1595205	PHEGAS329	Yes	2016
ERS1595178	PHEGAS330	Yes	2016
ERS1595179	PHEGAS331	Yes	2016
ERS1595207	PHEGAS332	Yes	2016
ERS1595208	PHEGAS333	Yes	2016
ERS1595210	PHEGAS335	Yes	2016
ERS1595186	PHEGAS336	Yes	2016
ERS1595183	PHEGAS337	Yes	2016
ERS1595213	PHEGAS339	Yes	2016
ERS1595215	PHEGAS340	Yes	2016
ERS1595217	PHEGAS341	Yes	2016
ERS1595220	PHEGAS343	Yes	2016
ERS1595222	PHEGAS344	Yes	2016
ERS1595224	PHEGAS345	Yes	2016
ERS1595225	PHEGAS346	Yes	2016 [L]
ERS1595227	PHEGAS347	Yes	2016
ERS1595229	PHEGAS348	Yes	2016
ERS1595231	PHEGAS349	Yes	2016
ERS1595232	PHEGAS350	Yes	2016
ERS1595234	PHEGAS351	Yes	2016
ERS1595235	PHEGAS352	Yes	2016
ERS1595237	PHEGAS353	Yes	2016
ERS1595239	PHEGAS354	Yes	2016
ERS1595241	PHEGAS355	Yes	2016[L]
ERS1595242	PHEGAS356	Yes	2016[L]
ERS1595244	PHEGAS357	Yes	2016
ERS1595246	PHEGAS358	Yes	2016
ERS1595248	PHEGAS359	Yes	2016
ERS1595250	PHEGAS360	Yes	2016
ERS1595251	PHEGAS361	Yes	2016
ERS1595253	PHEGAS362	Yes	2016[W]

ERS1595257	PHEGAS364	Yes	2016[L]
ERS1595260	PHEGAS366	Yes	2016
ERS1595261	PHEGAS367	Yes	2016
ERS1595263	PHEGAS368	Yes	2016[L]
ERS1595266	PHEGAS370	Yes	2016
ERS1595274	PHEGAS374	Yes	2016
ERS1595276	PHEGAS375	Yes	2016
ERS1595279	PHEGAS377	Yes	2016
ERS1595280	PHEGAS378	Yes	2016
ERS1595282	PHEGAS379	Yes	2016
ERS1595284	PHEGAS380	Yes	2016
ERS1595285	PHEGAS381	Yes	2016
ERS1595289	PHEGAS383	Yes	2016
ERS1595290	PHEGAS384	Yes	2016
ERS1595292	PHEGAS385	No	2016
ERS1595293	PHEGAS386	Yes	2016
ERS1595295	PHEGAS387	Yes	2016
ERS1595297	PHEGAS388	Yes	2016
ERS1595298	PHEGAS389	Yes	2016[W]
ERS1595300	PHEGAS390	Yes	2016
ERS1595302	PHEGAS391	Yes	2016
ERS1595305	PHEGAS393	Yes	2016
ERS1595307	PHEGAS394	No	2016
ERS1595308	PHEGAS395	Yes	2016
ERS1594996	PHEGAS397	Yes	2016
ERS1594997	PHEGAS398	Yes	2016
ERS1594999	PHEGAS400	Yes	2016
ERS1595000	PHEGAS401	Yes	2016
ERS1595001	PHEGAS402	Yes	2016
ERS1595002	PHEGAS403	Yes	2016
ERS1595005	PHEGAS406	Yes	2016
ERS1595007	PHEGAS408	Yes	2016
ERS1595008	PHEGAS409	Yes	2016
ERS1595009	PHEGAS410	Yes	2016
ERS1595010	PHEGAS411	Yes	2016[L]
ERS1595011	PHEGAS412	Yes	2016
ERS1595012	PHEGAS413	Yes	2016
ERS1595013	PHEGAS414	Yes	2016
ERS1595014	PHEGAS415	Yes	2016

ERS1595015	PHEGAS416	Yes	2016
ERS1595016	PHEGAS417	Yes	2016
ERS1595017	PHEGAS418	Yes	2016
ERS1595018	PHEGAS419	Yes	2016
ERS1595019	PHEGAS420	Yes	2016
ERS1595021	PHEGAS422	Yes	2016
ERS1595022	PHEGAS423	Yes	2016
ERS1595023	PHEGAS424	Yes	2016
ERS1595024	PHEGAS425	Yes	2016
ERS1595025	PHEGAS426	Yes	2016
ERS1595027	PHEGAS428	Yes	2016
ERS1595028	PHEGAS429	Yes	2016
ERS1595029	PHEGAS430	Yes	2016
ERS1595030	PHEGAS431	Yes	2016
ERS1595031	PHEGAS432	Yes	2016[L]
ERS1595032	PHEGAS433	Yes	2016
ERS1595033	PHEGAS434	Yes	2016[W]
ERS1595034	PHEGAS435	Yes	2016
ERS1595035	PHEGAS436	Yes	2016
ERS1595036	PHEGAS437	Yes	2016[W]
ERS1595037	PHEGAS438	Yes	2016
ERS1595038	PHEGAS439	Yes	2016[L]
ERS1595039	PHEGAS440	Yes	2016
ERS1595040	PHEGAS441	Yes	2016[L]
ERS1595041	PHEGAS442	Yes	2016
ERS1595042	PHEGAS443	Yes	2016
ERS1595043	PHEGAS444	No	2016
ERS1595045	PHEGAS445	Yes	2016
ERS1595046	PHEGAS446	Yes	2016
ERS1595048	PHEGAS447	Yes	2016
ERS1595049	PHEGAS448	Yes	2016
ERS1595051	PHEGAS449	Yes	2016
ERS1595053	PHEGAS450	Yes	2016
ERS1595054	PHEGAS451	Yes	2016[L]
ERS1595058	PHEGAS453	Yes	2016
ERS1595059	PHEGAS454	Yes	2016
ERS1595061	PHEGAS455	Yes	2016
ERS1595062	PHEGAS456	Yes	2016
ERS1595064	PHEGAS457	Yes	2016

ERS1595066	PHEGAS458	Yes	2016
ERS1595067	PHEGAS459	Yes	2016
ERS1595069	PHEGAS460	Yes	2016
ERS1595071	PHEGAS461	Yes	2016[W]
ERS1595072	PHEGAS462	Yes	2016
ERS1595074	PHEGAS463	Yes	2016
ERS1595076	PHEGAS464	Yes	2016
ERS1595077	PHEGAS465	Yes	2016
ERS1595079	PHEGAS466	Yes	2016
ERS1595081	PHEGAS467	Yes	2016
ERS1595082	PHEGAS468	Yes	2016
ERS1595084	PHEGAS469	Yes	2016
ERS1595086	PHEGAS470	Yes	2016
ERS1595088	PHEGAS471	No	2016
ERS1595089	PHEGAS472	Yes	2013
ERS1595091	PHEGAS473	Yes	2013[W]
ERS1595093	PHEGAS474	Yes	2013
ERS1595094	PHEGAS475	Yes	2016
ERS1595096	PHEGAS476	Yes	2016
ERS1595113	PHEGAS477	Yes	2016
ERS1595114	PHEGAS478	Yes	2016

[L] denotes isolates from London
[W] denotes isolates from Wales
Sequences were used in Figures 4, 5, S3-5

Supplementary table 4: Previously sequenced invasive and sterile site *emm1 S. pyogenes* strains isolated from UK, March-May 2014-2015 used in comparative phylogenetic analysis (n=127)[1]

ENA	Isolate	Year
ERR1735326	GASEMM0282	2014
ERR1734716	GASEMM0286	2014
ERR1734694	GASEMM0288	2014
ERR1734317	GASEMM0296	2014
ERR1734401	GASEMM0321	2014
ERR1734888	GASEMM0325	2014
ERR1732999	GASEMM0330	2014
ERR1732882	GASEMM0341	2014
ERR1734868	GASEMM0350	2014
ERR1732487	GASEMM0351	2014
ERR1734992	GASEMM0398	2014
ERR1733933	GASEMM0407	2014
ERR1733306	GASEMM0408	2014
ERR1735043	GASEMM0423	2014
ERR1734816	GASEMM0500	2014[L]
ERR1734756	GASEMM0505	2014
ERR1735004	GASEMM0506	2014
ERR1732742	GASEMM0507	2014
ERR1733329	GASEMM0511	2014
ERR1732864	GASEMM0525	2014
ERR1733180	GASEMM0545	2014
ERR1732631	GASEMM0557	2014
ERR1733571	GASEMM0567	2014
ERR1735458	GASEMM0617	2014
ERR1734831	GASEMM0623	2014[L]
ERR1733026	GASEMM0628	2014
ERR1733140	GASEMM0629	2014
ERR1732584	GASEMM0632	2014
ERR1734543	GASEMM0633	2014
ERR1734818	GASEMM0659	2014
ERR1734600	GASEMM0699	2014
ERR1733043	GASEMM0700	2014[L]
ERR1733748	GASEMM0706	2014
ERR1735013	GASEMM0785	2014
ERR1733362	GASEMM0832	2014

ERR1732979	GASEMM0835	2014
ERR1733107	GASEMM0848	2014
ERR1735413	GASEMM0883	2014
ERR1734302	GASEMM0902	2014
ERR1734359	GASEMM0936	2014
ERR1734874	GASEMM0947	2014
ERR1733307	GASEMM0964	2014
ERR1734282	GASEMM1017	2014
ERR1732733	GASEMM1027	2014
ERR1732554	GASEMM1030	2014
ERR1733246	GASEMM1154	2014[L]
ERR1734285	GASEMM1246	2014
ERR1734445	GASEMM2528	2015
ERR1735332	GASEMM2530	2015
ERR1732875	GASEMM2538	2015
ERR1734262	GASEMM2545	2015[L]
ERR1734038	GASEMM2555	2015
ERR1733093	GASEMM2562	2015
ERR1732547	GASEMM2565	2015
ERR1732552	GASEMM2571	2015
ERR1733202	GASEMM2578	2015
ERR1734314	GASEMM2581	2015
ERR1732721	GASEMM2588	2015
ERR1732654	GASEMM2589	2015
ERR1734593	GASEMM2634	2015
ERR1734957	GASEMM2644	2015
ERR1734174	GASEMM2659	2015
ERR1732585	GASEMM2667	2015
ERR1733511	GASEMM2670	2015
ERR1732667	GASEMM2677	2015
ERR1732894	GASEMM2686	2015
ERR1732670	GASEMM2687	2015
ERR1735158	GASEMM2701	2015
ERR1732988	GASEMM2703	2015
ERR1733986	GASEMM2721	2015
ERR1733384	GASEMM2727	2015
ERR1733241	GASEMM2738	2015
ERR1735044	GASEMM2754	2015
ERR1734897	GASEMM2755	2015

ERR1733013	GASEMM2757	2015
ERR1734726	GASEMM2758	2015
ERR1733235	GASEMM2764	2015[L]
ERR1733438	GASEMM2765	2015
ERR1735390	GASEMM2777	2015
ERR1732682	GASEMM2781	2015
ERR1733038	GASEMM2789	2015
ERR1733723	GASEMM2799	2015
ERR1734009	GASEMM2804	2015
ERR1733032	GASEMM2810	2015[L]
ERR1732766	GASEMM2814	2015
ERR1732756	GASEMM2815	2015
ERR1733919	GASEMM2822	2015
ERR1735263	GASEMM2828	2015
ERR1734229	GASEMM2839	2015
ERR1732779	GASEMM2843	2015
ERR1734880	GASEMM2844	2015
ERR1732792	GASEMM2854	2015
ERR1733155	GASEMM2857	2015
ERR1734254	GASEMM2859	2015
ERR1734463	GASEMM2862	2015
ERR1733051	GASEMM2877	2015
ERR1733227	GASEMM2879	2015
ERR1732439	GASEMM2899	2015
ERR1733240	GASEMM2907	2015
ERR1733629	GASEMM2909	2015
ERR1732524	GASEMM2917	2015
ERR1734790	GASEMM2921	2015[L]
ERR1733469	GASEMM2927	2015
ERR1735407	GASEMM2929	2015
ERR1734828	GASEMM2934	2015
ERR1732777	GASEMM2938	2015
ERR1732705	GASEMM2943	2015
ERR1734841	GASEMM2944	2015
ERR1734968	GASEMM2954	2015
ERR1734769	GASEMM2961	2015
ERR1735365	GASEMM2963	2015
ERR1732647	GASEMM2969	2015
ERR1734520	GASEMM2970	2015

ERR1732863	GASEMM2971	2015
ERR1733744	GASEMM2972	2015
ERR1733348	GASEMM2978	2015
ERR1734914	GASEMM2982	2015
ERR1734240	GASEMM2983	2015
ERR1734571	GASEMM2984	2015
ERR1732580	GASEMM2987	2015
ERR1734984	GASEMM2995	2015
ERR1733904	GASEMM3002	2015
ERR1734523	GASEMM3007	2015
ERR1735244	GASEMM3017	2015
ERR1733279	GASEMM3019	2015
ERR1733678	GASEMM3027	2015
ERR1735194	GASEMM3028	2015

[1]Sequences were used in the analysis for Figures 4, 5, S3-5
[L] denotes London

Supplementary table 5. Genome sequences of *emm*1 isolates used for longitudinal UK analysis and comparative global phylogenetic analysis

Region of origin	Reference
UK (n=1240)	This study n=581; n=610[1]; n=25[2]; n=24[3]
North America (n=720)	n=316[4]; n=404 (USA, n=381; Canada n=23)[5]
Nordic regions (n=806)	n=806 (Denmark, n=269; Sweden, n=263; Finland, n=204; Iceland, n=16; Norway, n=54)[5]
South-east Asia (n=34)	n=34 (Hong Kong, n=18; China, n=16)[6]

Sequences of uncertain quality or origin were not used in the analysis.
Sequences from Kapatai *et al*[1] included UK isolates from a 14 month continuous period 2014-2015
Sequences from Turner *et al*[2,3] included isolates from England 2007-2012
Sequences from Nasser *et al*.[5] prior to 2005 were not included.

Supplementary Methods

Isolate sampling. Non-invasive *S. pyogenes* isolates were collected throughout each year 2009-2013. Dwindling in sample submissions 2009-2013, linked to practice and UK guideline change, rendered whole year collection operationally impractical. An unexpected increase in sample submissions in February 2014 and alert regarding increasing scarlet fever notifications prompted collection during a fixed period March-May 2014 for operational reasons, and to ensure robust protocol adherence. For consistency, collection of non-invasive strains was undertaken in March-May of subsequent years 2015 and 2016.

Invasive isolates 2013-2016 were collected throughout each year by the Reference laboratory and all were *emm*-genotyped; invasive *emm*1 isolates for sequencing were selected from March-May 2013 and March-May 2016 for consistency with the non-invasive strain collection and to focus on the season with maximum notifications of invasive disease. Sequences from 2014 and 2015 invasive isolates were previously reported[1]. Phylogenetic analysis and comparison of 552 sterile site invasive *emm*1 isolates from 2013-2016 was limited to cases arising in March-May.

***Emm* genotyping.** *S. pyogenes emm* sequencing was performed according to the protocol of the Centers for Diseases Control and Prevention (www.cdc.gov/ncidod/biotech/strep/protocol_emmtype).

DNA extraction and genome sequencing. Genomic DNA was extracted as described previously[7] or using Qiasymphony (Qiagen, Germany)[8]. Multiplexed DNA library preparation was conducted according to the Illumina protocol and sequencing was performed on an Illumina HiSeq 2000 (Illumina, USA) with 100-cycle paired-end runs. All genome sequence data generated in this study have been submitted to the European Nucleotide Archive (ENA) (www.ebi.ac.uk/ena) under the accession numbers listed in Supplementary Tables S2 and S3.

Genomic analysis. Multilocus sequence typing by blast from de novo assemblies against PubMLST[9] was performed for all sequenced isolates, and e*mm*-types were confirmed by *in-silico* PCR (https://www.ebi.ac.uk/about/vertebrate-genomics/software/ipcress-manual) followed by BLAST analysis using the CDC *emm*-typing database (https://www2a.cdc.gov/ncidod/biotech/strepblast.asp).
SMALT (Wellcome Trust Sanger Institute) was used to map reads to the designated *emm*1 reference strain MGAS5005 (NC_007297). Concatenated SNPs in the core genome were used to construct a maximum-likelihood tree using RAxML[10]. Phylogenetic trees were generated and annotated using FigTree (http://tree.bio.ed.ac.uk/software/figtree/) and Dendroscope[11]. The global genetic context of strains collected during this study was elucidated by comparison with other published *S. pyogenes emm*1 genomes collected from the USA, Europe and Asia[1-6].
Pan-genome of 135 non-invasive *emm*1 isolates was queried using Roary (http://sanger-pathogens.github.io/Roary/)[12]. Regions of SNPs clustering and potential areas of recombination were investigated using Gubbins.[13]
All 135 sequenced non-invasive *emm*1 isolates were tested for the presence and absence of acquired antimicrobial resistance genes using ARIBA[14] and using the SRST2 database ARGANNOT[15].

Quantitative real-time PCR. RNA was extracted from *S. pyogenes* following 6 hours culture (37°C, 5% $C0_2$) and converted to cDNA following DNase treatment with TurboDNAfree (Ambion, Cambridgeshire UK) as described previously[16]. qrtRT-PCR was carried out for the *speA* gene (primers: *speA* F: 5'-GAGGGGTAACAAATCATGAAGG-3', *speA* R: 5'- TCAAATGATAGGCTTTGGATACC-3'), and expression data normalized to that of *proS* (primers: *proS* F: 5'-TGAGTTTATTATGAAAGACGGCTATAGTTTC-3', *proS* R: 5'-AATAGCTTCGTAAGCTTGACGATAATC-3') using a standard curve method as described previously[16].

***S. pyogenes* supernatant preparation and SpeA western blot.** *S. pyogenes* was cultured overnight in Todd-Hewitt Broth and supernatants were 0.2 μM filtered (Sartorius) then 5× concentrated using 10 kDa cutoff spin columns (Amicon). Total protein content of each sample was quantified using NanoDrop One

spectrophotometer (ThermoFisher). SDS-PAGE and western blotting were carried out as described previously[17]. SpeA concentrations in culture supernatants were quantified by densitometry by comparison with a standard curve of known concentrations of full length rSPEA (31kD with His-tag). Blots were probed with anti-rSpeA rabbit serum raised against a 17kD truncated rSPEA (1:1000) as described previously[18] and goat-anti rabbit-HRP secondary antibody (ThermoScientific). Blots were developed and densitometry performed using a Li-COR Odyssey Clx imaging system.

Reference List for Supplementary Material

1. Kapatai G, Coelho J, Platt S, Chalker VJ. Whole genome sequencing of group A Streptococcus: development and evaluation of an automated pipeline for emmgene typing. *PeerJ* 2017; **5**: e3226.
2. Turner CE, Bedford L, Brown NM, et al. Community outbreaks of group A Streptococcus revealed by genome sequencing. *Sci Rep* 2017; **7**(1): 8554.
3. Turner CE, Dryden M, Holden MT, et al. Molecular analysis of an outbreak of lethal postpartum sepsis caused by Streptococcus pyogenes. *J Clin Microbiol* 2013; **51**(7): 2089-95.
4. Chochua S, Metcalf BJ, Li Z, et al. Population and Whole Genome Sequence Based Characterization of Invasive Group A Streptococci Recovered in the United States during 2015. *MBio* 2017; **8**(5).
5. Nasser W, Beres SB, Olsen RJ, et al. Evolutionary pathway to increased virulence and epidemic group A Streptococcus disease derived from 3,615 genome sequences. *Proc Natl Acad Sci U S A* 2014; **111**(17): E1768-76.
6. Ben Zakour NL, Davies MR, You Y, et al. Transfer of scarlet fever-associated elements into the group A Streptococcus M1T1 clone. *Sci Rep* 2015; **5**: 15877.
7. Pospiech A, Neumann B. A versatile quick-prep of genomic DNA from gram-positive bacteria. *Trends Genet* 1995; **11**(6): 217-8.
8. Chalker V, Jironkin A, Coelho J, et al. Genome analysis following a national increase in Scarlet Fever in England 2014. *BMC Genomics* 2017; **18**(1): 224.
9. Page AJ. Multilocus sequence typing by blast from de novo assemblies against PubMLST. *The Journal of Open Source Software* 2016; **8**(1).
10. Stamatakis A. RAxML version 8: a tool for phylogenetic analysis and post-analysis of large phylogenies. *Bioinformatics* 2014; **30**(9): 1312-3.
11. Huson DH, Richter DC, Rausch C, Dezulian T, Franz M, Rupp R. Dendroscope: An interactive viewer for large phylogenetic trees. *BMC Bioinformatics* 2007; **8**: 460.
12. Page AJ, Cummins CA, Hunt M, et al. Roary: rapid large-scale prokaryote pan genome analysis. *Bioinformatics*. 2015;**31**:3691–3693.
13. Croucher NJ, Page AJ, Connor TR, et al. Rapid phylogenetic analysis of large samples of recombinant bacterial whole genome sequences using Gubbins. *Nucleic Acids Res*. 2015;**43**:e15.
14. Hunt M, Mather AE, Sánchez-Busó L, et al. ARIBA: rapid antimicrobial resistance genotyping directly from sequencing reads. *Microb Genom*. 2017;**3**:e000131.
15. Inouye M, Dashnow H, Raven LA, et al. SRST2: Rapid genomic surveillance for public health and hospital microbiology labs. *Genome Med*. 2014;**6**:90.
16. Turner CE, Kurupati P, Jones MD, Edwards RJ, Sriskandan S. Emerging role of the interleukin-8 cleaving enzyme SpyCEP in clinical Streptococcus pyogenes infection. *J Infect Dis* 2009; **200**(4): 555-63.
17. Lynskey NN, Goulding D, Gierula M, et al. RocA truncation underpins hyper-encapsulation, carriage longevity and transmissibility of serotype M18 group A streptococci. *PLoS Pathog* 2013; **9**(12): e1003842.
18. Sriskandan S, Unnikrishnan M, Krausz T, Cohen J. Molecular analysis of the role of streptococcal pyrogenic Exotoxin A (SPEA) in invasive soft-tissue infection resulting from Streptococcus pyogenes. *Mol Microbiol* 1999; **33**(4): 778-90.
